# Engineered N-doped graphene quantum dots/CoFe_2_O_4_ spherical composites as a robust and retrievable catalyst: fabrication, characterization, and catalytic performance investigation in microwave-assisted synthesis of quinoline-3-carbonitrile derivatives†

**DOI:** 10.1039/d1ra05739a

**Published:** 2021-10-27

**Authors:** Pouria Babaei, Javad Safaei-Ghomi

**Affiliations:** Department of Organic Chemistry, Faculty of Chemistry, University of Kashan Kashan 87317-51167 I. R. Iran safaei@kashanu.ac.ir

## Abstract

Nitrogen-doped graphene quantum dots (N-GQDs), which are less than 10 nm in size, are an interesting member of the nanocarbon materials family. N-GQDs nanostructures have been broadly applied in various fields, such as drug-gene delivery systems, photocatalytic reactions, and catalysts, owing to their unique properties. However, N-GQDs have rarely been introduced as a catalyst in organic synthesis. Herein, CoFe_2_O_4_ nanocomposites with diverse morphologies are fabricated in various conditions (*e.g.* green routes, various pH adjusters, temperatures). Due to further active sites on the surface of the nanocomposites, morphology engineering can be effective in catalytic activities. Following the synthesis, the catalytic activity of the engineered CoFe_2_O_4_ nanocomposites was screened, and it presented the order of spherical > rod > prism > cubic. The uniform spherical morphology provides more accessible active sites. Then, the novel nano-sized N-GQDs/CoFe_2_O_4_ magnetic spherical composite was readily fabricated by a green, low-cost, and easy hydrothermal route. The engineered composite was applied as an efficient magnetic nanocatalyst for the MW-assisted one-pot synthesis of new and known quinoline-3-carbonitrile derivatives (83–96%) in the shortest reaction time (60–90 s). Furthermore, the green route, easy separation of the nanocatalyst, and reusability (7 runs) without noticeable loss of catalytic efficiency are other advantages.

## Abstract 2

Sustainable fabrication of spherical N-GQDs/CoFe_2_O_4_ nanocomposites as a novel magnetically retrievable catalyst for the synthesis of quinoline-3-carbonitrile derivatives has been developed.

## Graphical abstract

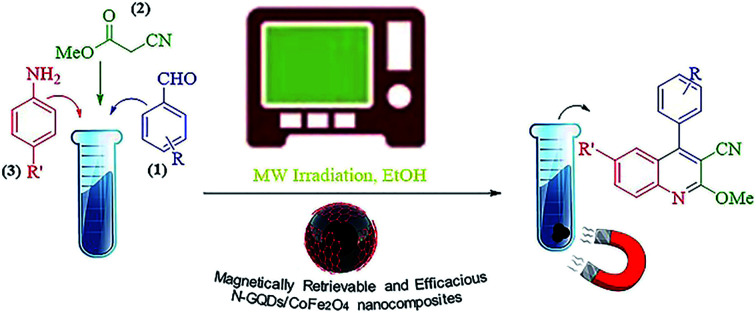

## 1. Introduction

In recent decades, numerous categories of nano-sized carbon materials, such as fullerene, carbon nanohorns, carbon quantum dots (CQDs), and graphene quantum dots (GQDs), have been developed owing to their exceptional physicochemical attributes.^1,2^ GQDs, which are a new type of carbon nanostructure, reveal many interesting attributes. These properties have their origins in their distinguished structure features.^3,4^ Also, they can be used for drug delivery, biosensors, catalyst activity, *etc.*^5^ Generally, GQDs can be fabricated by applying two approaches: “top-down” link broken various carbon structures, and “bottom-up” preparation from polymers or organic compounds such as citric acid.^6,7^ Furthermore, the presence of functional groups (*e.g.* hydroxyl and carboxyl) on the edges of GQD structures can be applied as bonding agents to the substrate or coating materials. On the other hand, some reports indicated that doping of GQDs with heteroatoms, such as nitrogen, sulfur, and phosphorus, can successfully increase the number of active sites owing to the modulated bandgap.^8,9^ Besides, retrieval of these carbon nanostructures from the reaction environment can be modified with diverse materials, such as nanomaterials, organic compounds, and polymers.^10–12^ Among them, magnetic nanostructures are of sharp interest to investigators due to their outstanding magnetic attributes.^13^ Moreover, structural comparative studies indicate that the magnetic attributes of metal oxide nanocomposites are superior to those of the bulk form.^14^ On the other hand, the choice of catalyst for economical production *via* a green route depends on various parameters, such as activity, selectivity, and stability.^15^ Hence, the N-GQDs/CoFe_2_O_4_ nanocomposite is one of the most practical soft-magnetic compounds due to its low price, nontoxicity, chemical stability, and high resistance to erosion.^16,17^

**Fig. 1:**
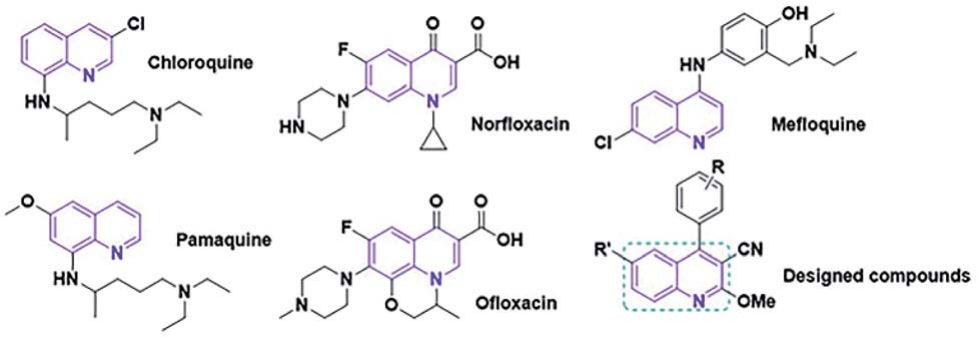
The quinoline scaffold.

The quinoline framework is known as an interesting aromatic heterocyclic organic compound. In organic synthesis, quinoline and its derivatives play chief roles because of their pharmacological and biological attributes^18,19^ (*e.g.* anticancer, anti-inflammatory, antimalarial, antimicrobial).^20–22^ Several medication compounds with quinoline scaffolds are revealed in Fig. 1.

Furthermore, various procedures have been studied for the preparation of quinoline derivatives, such as tandem reactions in the presence of NaOH,^23^ solvent-free conditions,^24^ various catalysts in different solvents,^25,26^ modified Skraup methods,^27^ the Doebner–Miller reaction,^28^ and Friedlander synthesis.^29^ Also, various metrics have been exploited to achieve green chemistry, such as using recoverable catalysts, elimination or minimization of hazardous solvents, and decreasing the number of reagents.^30,31^ Among these metrics, energy conservation has played a remarkable role in green synthesis. The microwave irradiation (MW) approach has been characterized as an alternative energy source and green method. Suppression of by-products, ameliorated yields, and very short reaction times can be mentioned as the other notable features of this procedure. Hence, microwave-assisted (MW-assisted) organic synthesis has had a significant impact on organic synthesis as well as on the way that researchers approach it.^32^

**Scheme 1:**
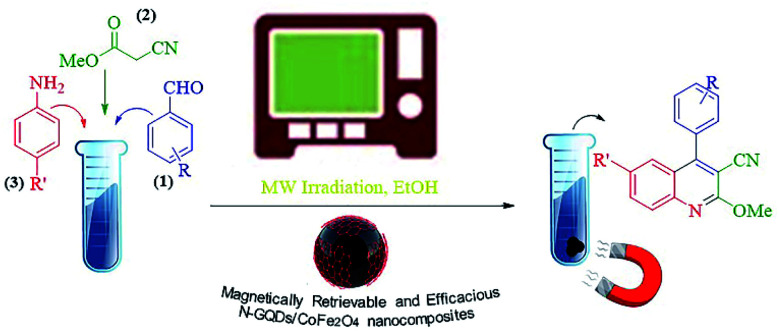
MW-assisted synthesis of quinoline-3-carbonitrile frameworks.

The main goal of this study, the design, formation, and identification of N-GQDs/CoFe_2_O_4_ nanocomposites as a novel, stable, efficient, and recoverable catalyst, is reported for the synthesis of quinoline-3-carbonitrile derivatives, with investigation of the main factors which enable control of its catalytic performance. The catalytic performance results have shown that spherical morphology had the most catalytic activity due to the large surface area of the CoFe_2_O_4_ spherical nanocomposite. As a result, we devoted our efforts to investigating the MW-assisted preparation of quinoline-3-carbonitrile derivatives in ethanol as a green solvent in the presence of the N-GQDs/CoFe_2_O_4_ spherical nanocomposite. The N-GQDs/CoFe_2_O_4_ spherical nanocomposite was prepared as an efficacious and magnetically retrievable nanocatalyst *via* the hydrothermal route. As illustrated in Scheme 1, quinoline-3-carbonitrile derivatives were designed and synthesized by a MW-assisted three-component reaction of methyl 2-cyanoacetate, various aromatic aldehydes, and different aromatic amines in the presence of the N-GQDs/CoFe_2_O_4_ nanocomposites.

## 2. Experimental

### 2.1. Materials and general procedures

All chemicals were of analytical grade, obtained from Scharlau and Merck, and used without extra purification. To monitor the progress of the reactions, we used TLC (pre-coated silica gel 60 F254, Merck), and all products were characterized by melting point and FT-IR, ^1^H NMR, and ^13^C NMR spectroscopy analysis. The melting points were obtained using an Electro-Thermal 9200 instrument and are not corrected. FT-IR spectra were measured using an FT-IR instrument (KBr pellets, Nicolet Magna 550 FT-IR spectrometer). ^1^H and ^13^C NMR spectra were recorded in CDCl_3_ on a Bruker spectrometer at 400 and 100 MHz, respectively (internal reference: TMS). Besides, the prepared CoFe_2_O_4_@N-GQDs magnetic nanocomposite was characterized by TEM, FE-SEM, XRD, FT-IR, Raman, AFM, EDS mapping, TGA, and VSM analysis.

### 2.2. Fabrication of the CoFe_2_O_4_ spherical nanocomposite

Initially, the same molar ratio of Fe^2+^ : Fe^3+^ (from FeSO_4_·7H_2_O and Fe(NO_3_)_3_·9H_2_O, respectively) was dissolved completely in 35 ml deionized water at ambient temperature. After that, as-prepared Co^2+^ solution (0.3 g Co(NO_3_)_2_·6H_2_O in 10 ml deionized water) was added to the above solution by dropping. Under continuous stirring, the previously prepared alkaline aqueous solution was added dropwise to the final solution to bring the pH to 12 (see Table 1). The solution directly became dark after addition of the alkaline solution, and it was stirred at 25 °C (15 min). Eventually, the whole mixture was placed in a 150 ml Teflon-lined stainless steel autoclave and transferred to an electric oven at 150 °C for 12 hours under hydrothermal conditions. The black solid was collected by an external magnetic field. The prepared CoFe_2_O_4_ spherical nanostructures were washed with dry ethanol and dried in a vacuum overnight at 90 °C.

**Table 1:** Applied conditions for the fabrication of the CoFe_2_O_4_ spherical nanocomposite

No.	Synthesis route	pH adjuster	Temp. (°C)	Time	Power (W)	Shape
1	Hydrothermal	NaOH	180	15 h	—	Spherical/hemi-hexagonal
2	Hydrothermal	NaOH	150	15 h	—	Spherical
3	Hydrothermal	NaOH	150	15 h	—	Spherical
4	Hydrothermal	NH_3_	150	15 h	—	Prism
5	MW irradiation	NaOH	150	7 min	600	Cubic
6	MW irradiation	NH_3_	150	7 min	600	Rod

### 2.3. Fabrication of the N-GQDs/CoFe_2_O_4_ spherical nanocomposite

**Scheme 2:**
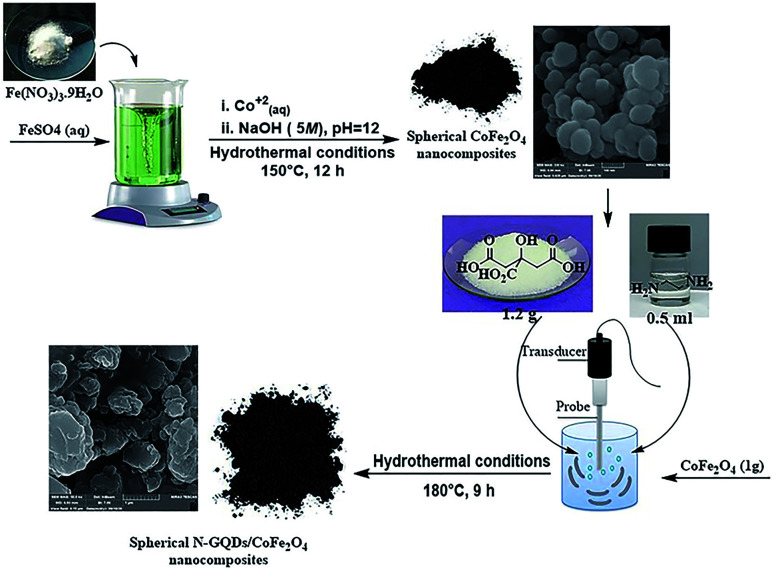
Fabrication of the N-GQDs/CoFe_2_O_4_ spherical nanocomposite.

The hydrothermal method is the foremost common route for the mass production of GQDs.^33^ A mixture of citric acid (CA, 1 g), ethylenediamine (en, 0.4 ml), and ultrapure water (50 ml) was stirred for 2 minutes at ambient temperature to form a clear homogeneous mixture.^34^ Then, the as-prepared CoFe_2_O_4_ nanocomposite (1 g) was poured into the above mixture and sonicated for 1 min to make a homogeneous mixture. Afterward, the mixture was placed in a 150 ml Teflon-lined stainless steel autoclave and placed in an electric oven at 180 °C for 9 hours under hydrothermal conditions. At completion, the resulting sediment was collected under an external magnetic field and washed with dry ethanol. The separated precipitate, finally, was dried at 60 °C for 24 hours under vacuum conditions (Scheme 2).

### 2.4. Synthesis of quinoline-3-carbonitriles under reflux conditions

A 25 ml round bottom flask was charged with the same molar ratio of various aryl aldehydes (1), methyl cyanoacetate (2), different substituted anilines (3), N-GQDs/CoFe_2_O_4_ (20 mg), and ethanol. Subsequently, the whole mixture was stirred under reflux conditions for the appropriate time. The reaction progress was monitored by TLC. After the completion of the reaction, the crude product was filtered and washed with cold dry ethanol to obtain the pure product.

### 2.5. Synthesis of quinoline-3-carbonitriles under microwave irradiation

A 25 ml round bottom flask was charged with the same molar ratio of various aryl aldehydes (1), methyl cyanoacetate (2), different substituted anilines (3), N-GQDs/CoFe_2_O_4_ (20 mg), and ethanol. The whole mixture was stirred for 1 minute at ambient temperature. After thorough mixing, the prepared mixture was exposed to microwave irradiation at 400 W. Upon completion of the reaction (monitored by TLC), the crude product was filtered and washed with cold dry ethanol to obtain the pure product. All products were characterized by ^1^H NMR, ^13^C NMR, and FT-IR spectroscopy.

## 3. Results and discussion

### 3.1. Characterization and structural studies of the spherical N-GQDs/CoFe_2_O_4_ nanocomposites

**Fig. 2:**
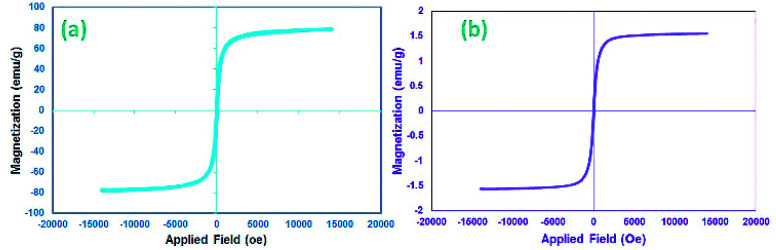
VSM curves of the (a) CoFe_2_O_4_ and (b) N-GQDs/CoFe_2_O_4_ magnetic nanocomposite.

Many research articles have been hitherto published about catalysts and catalytic activities. Also, catalytic performance has had a profound effect on the way chemists have chosen organic compound synthesis methods.^35^ Among these catalysts, heterogeneous magnetic catalysts have been significantly expanded because of their easy workup methods, facile fabrication, high permanence, and high thermal stability. Moreover, their magnetic properties are related to remarkable reusability.^36^ The magnetic attributes of the as-fabricated nanocomposites were measured by a vibrating sample magnetometer (VSM) at room temperature. The saturation magnetization (*M*_s_) value of the CoFe_2_O_4_ sphere nanocomposites is approximately 80 emu g^−1^, which is suggestive of powerful magnetic attributes (Fig. 2a). The CoFe_2_O_4_ spherical nanocomposites were decorated with N-GQDs nanostructures. The magnetic attributes were reduced sharply (Fig. 2b). This change reveals that this decline could be a result of the existence of a non-magnetic layer.^37^ Therefore, the nano-sized N-GQDs/CoFe_2_O_4_ magnetic composites were formed and could also be easily separated from the reaction mixture by an external magnet.

**Fig. 3:**
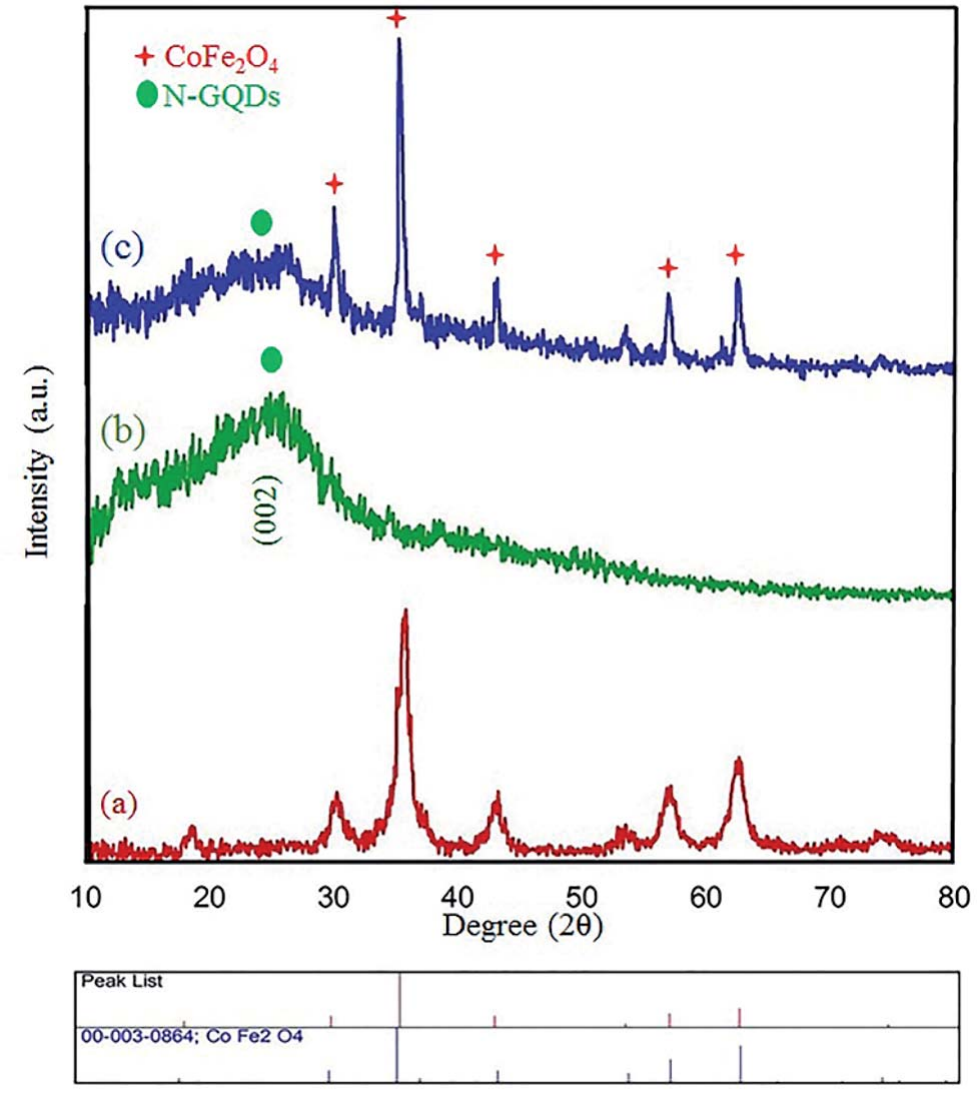
XRD patterns of the as-prepared CoFe_2_O_4_ (a), pure N-GQDs (b), and CoFe_2_O_4_/NGQDs nanocomposite (c).

The XRD technique can be used to identify the crystallinity of a composition, and it was used to investigate the purity of the as-prepared nanostructures.^38^ The XRD patterns of pure CoFe_2_O_4,_ pure N-GQDS, and the CoFe_2_O_4_/N-GQDs spherical nanostructures are shown in Fig. 3. As presented in Fig. 3a, the diffraction angles (2*θ*) for the magnetic nanocomposites that are evident at ∼30.4°, 35.7°, 38°, 43.4°, 53.8°, 57.3°, 62.9°, and 71.4° can be indexed to the (220), (311), (222), (400), (422), (333), (440), and (622) planes, respectively. The XRD results confirm the fabrication of CoFe_2_O_4_ nanocomposites with JCPDS card no. 003-0864.^39^ The crystalline size, which is determined by the Debye–Scherrer equation, was obtained as approximately 10 nm. The absence of extra diffraction lines in the XRD graph indicates the purity of the as-fabricated CoFe_2_O_4_ crystal. In the case of Fig. 3b, a broad peak at 2*θ* = 24° (002 plane) can be related to the amorphous structure of the N-GQDs.^40^ According to Fig. 3c, all Bragg peaks of the nano-sized CoFe_2_O_4_ composites and N-GQDs were seen at the same time, and the CoFe_2_O_4_/N-GQDs phase compositions did not change.

**Fig. 4:**
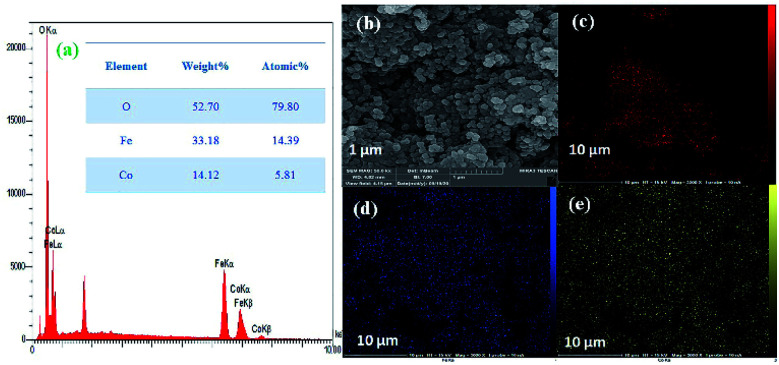
EDS analysis of the CoFe_2_O_4_ sphere nanocomposite (a) and surface elemental mapping-EDS of Co, Fe, and O elements (b–e).

**Fig. 5:**
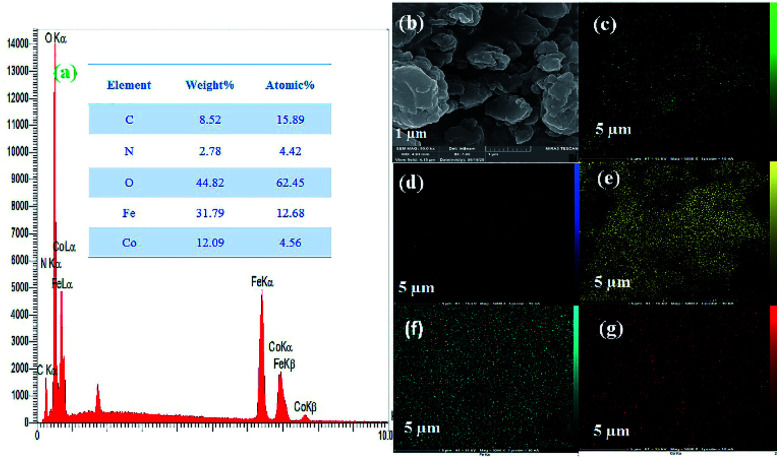
EDS analysis of the N-GQDs/CoFe_2_O_4_ nanocomposite (a) and surface elemental mapping-EDS of Co, Fe, O, C, and N elements (b–g).

Next, the purity of CoFe_2_O_4_ and the N-GQDs/CoFe_2_O_4_ nanocomposite was investigated by element distribution and elemental mapping analysis. The results denote that all the elements in the as-fabricated magnetic CoFe_2_O_4_ nanocomposite are Co (14.12%), Fe (33.18%), and O (52.70%), as shown in Fig. 4a. Also, the mapping-EDS results (Fig. 4b–e) depict the existence of elements in the CoFe_2_O_4_ nanocomposite. Besides, the final EDS results prove not only the fabrication of CoFe_2_O_4_ but also the presence of C (8.52%), and N (2.78%) in the N-GQDs/CoFe_2_O_4_ nanocomposite (Fig. 5a). From the results, it was concluded that all elements (C, N, O, Fe, and Co) are well distributed throughout the N-GQDs/CoFe_2_O_4_ nanocomposite (Fig. 5b–g).

**Fig. 6:**
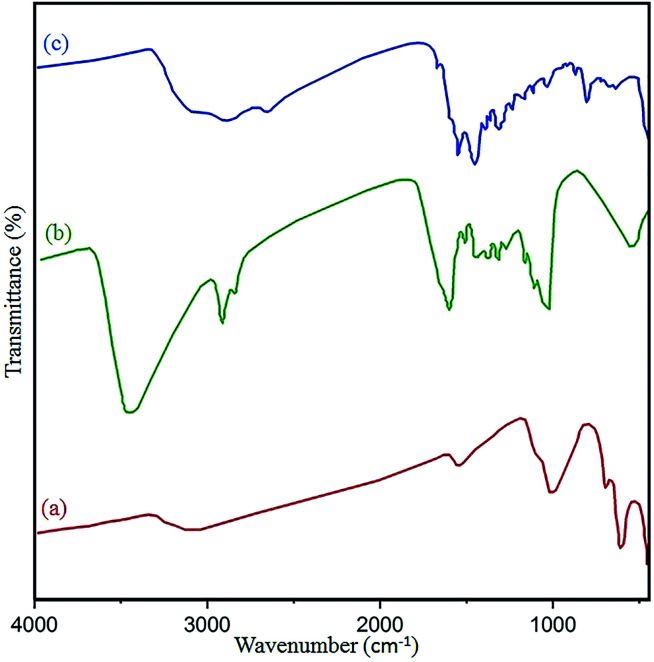
FT-IR spectra of the (a) as-prepared CoFe_2_O_4_, (b) pure N-GQDs, and (c) N-GQDs/CoFe_2_O_4_ nanocomposite.

The structure and surface functional groups of pristine N-GQDs and the CoFe_2_O_4_ magnetic nanocomposite were further probed by FT-IR spectroscopy. The absorption peaks at 1022 cm^−1^ and 590 cm^−1^ are related to Co–Fe and Fe–O, respectively. Also, the absorbance peaks at 1628 cm^−1^ and 3410 cm^−1^ were related to OH bending and stretching vibrations, respectively, due to absorbed H_2_O on the surface of the nanocomposite (Fig. 6a).^41^ In the case of N-GQDs, a peak located at 3385 cm^−1^ has been attributed to the vibration of –OH stretching. Also, absorbance peaks at approximately 3198 cm^−1^ and 2920 cm^−1^ confirm the =CH (sp^2^) and –CH(sp^3^) modes of the N-GQDs (Fig. 6b and c).^42^ Compared to the CoFe_2_O_4_ spectrum, the new bands at 1680 cm^−1^, 1550 cm^−1^, and 1388 cm^−1^ in the final spectrum correspond to the C=O, C=C, and C–O/C–N stretching vibrations of the GQDs, respectively.^43^ Therefore, the observed data confirm the formation of the N-GQDs/CoFe_2_O_4_ nanocomposite.

**Fig. 7:**
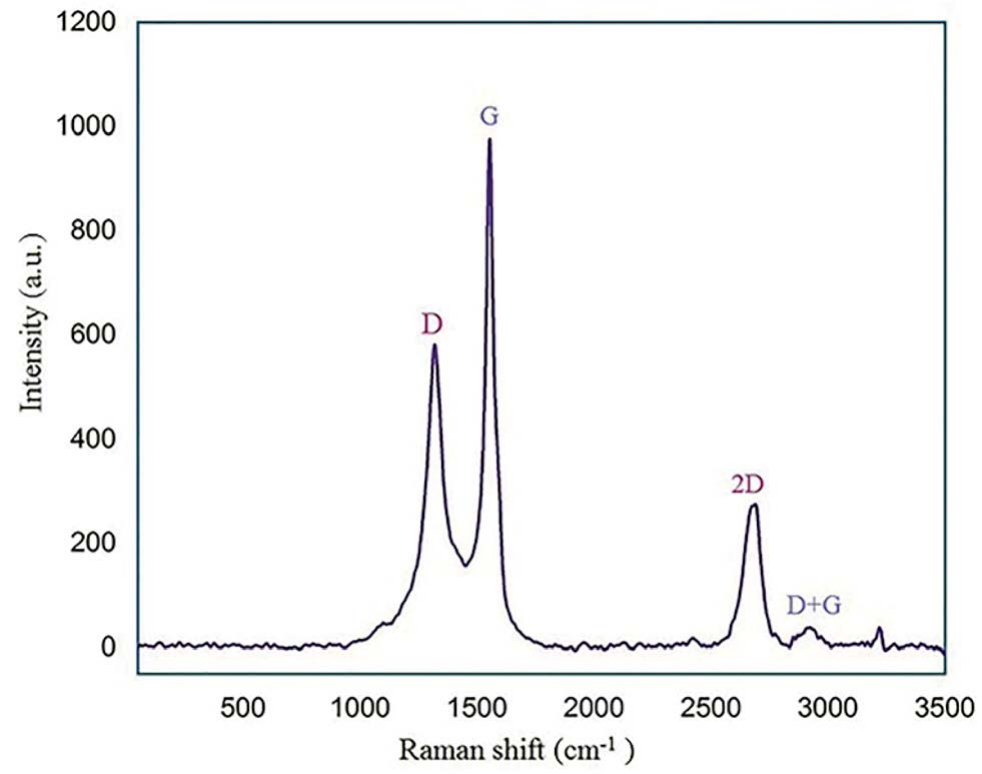
Raman spectrum of the N-GQDs/CoFe_2_O_4_ nanocomposite.

Raman spectroscopy is another identification technique to study carbon material structures. The Raman graph of GQDs usually displays D (disorder in C_sp_^2^) and G (first-order scattering of the stretching vibration for C_sp_^2^) bands at about 1350 cm^−1^ and 1585 cm^−1^, respectively.^44^ As shown in Fig. 7, the two characteristic peaks at approximately 1319 cm^−1^ and 1554 cm^−1^ correspond to the D and G bands, respectively. A peak at 2690 cm^−1^ and a broad weak peak around 2930 cm^−1^ are related to the 2D and D + G bands, respectively.^45^ According to the Raman spectrum, one can find that the nano-sized N-GQDs/CoFe_2_O_4_ composite was successfully formed.

**Fig. 8:**
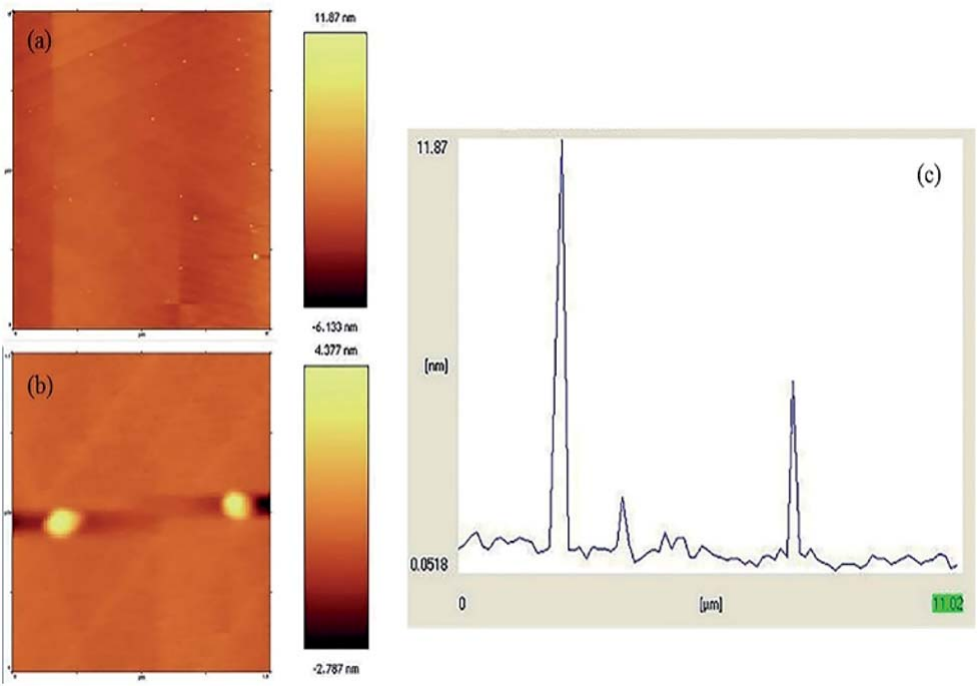
(a and b) AFM image of the N-GQDs/CoFe_2_O_4_ nanocomposite, and (c) the profile along the line in (a).

The formation of the N-GQDs was confirmed by their AFM topography image. GQDs are graphene sheets, and their lateral dimensions are less than 20 nm.^46^Fig. 8a and b depict the AFM image of the N-GQDs/CoFe_2_O_4_ nanocomposite, the sizes of which are less than 11 nm (Fig. 8c). Without a doubt, the significant conclusion to be drawn from the AFM image and profile is that the formation of the N-GQDs/CoFe_2_O_4_ nanocomposite was successful.

**Fig. 9:**
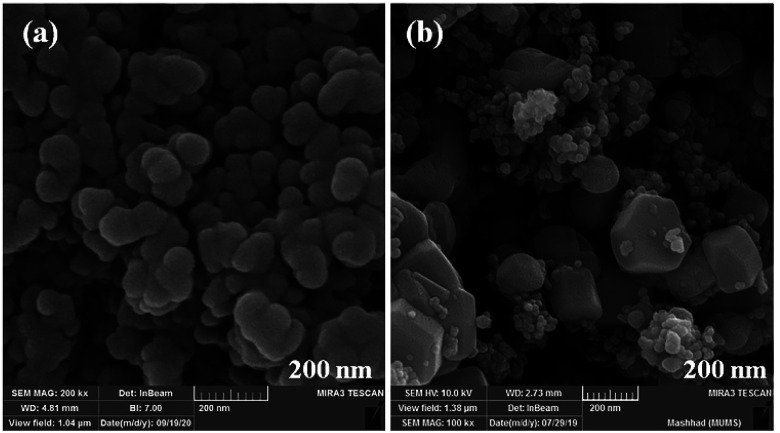
FE-SEM images of CoFe_2_O_4_ prepared *via* the hydrothermal route for 15 h at (a) 150 °C, and (b) 180 °C.

**Fig. 10:**
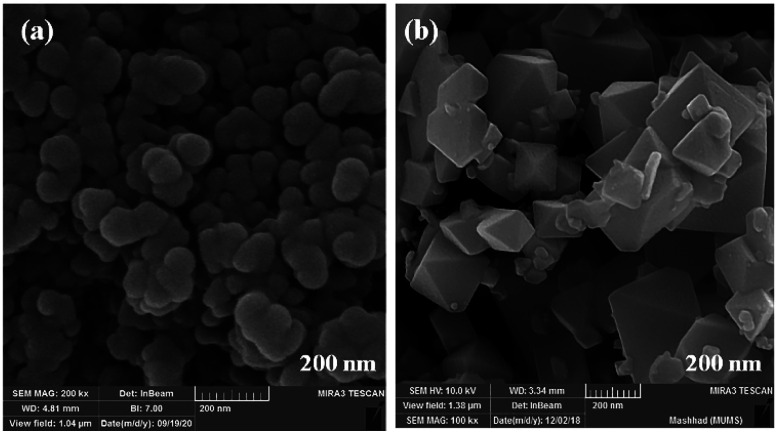
FE-SEM images of CoFe_2_O_4_ prepared *via* the hydrothermal route at 150 °C for 15 h in the presence of different pH adjusters: (a) NaOH and (b) NH_3_.

The size and morphology of particles play a key role in their catalytic activities. Besides, reaction time, temperature, and pH adjustment can remarkably affect the morphology and particle size.^47^ Therefore, hydrothermal treatment is a powerful tool for the investigation of these purposes.^48^ In this work, the FE-SEM technique was applied to study the particle size and morphology attributes. NaOH and NH_3_ were also used as pH adjusters. The FE-SEM images showed that the as-prepared CoFe_2_O_4_ nanocomposite could be recognized as having spherical shapes in the presence of NaOH at various temperatures (Fig. 9). The particle sizes of all the samples were measured by FE-SEM analysis.^49^ The average diameter of the nanocomposite was calculated to be about 55 nm. It can be observed from the FE-SEM images that many primary nanostructures with spherical shapes were converted to a prism-shaped morphology in the presence of NH_3_. The particle size was reported as approximately 80 nm (Fig. 10). Furthermore, in various studies, it was reported that spherical nanostructures possess higher surface areas and greater catalytic activities, whereas other morphologies indicated the opposite.^50^ To achieve this morphology, NaOH solution is an appropriate option to adjust the pH.

**Fig. 11:**
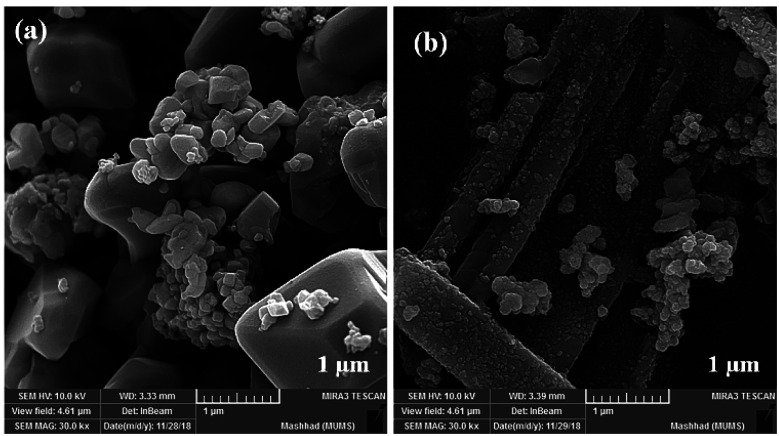
FE-SEM images of CoFe_2_O_4_ prepared *via* the MW method in the presence of (a) NaOH and (b) NH_3_.

Although the MW route is faster and simpler, no uniform nanostructure formation was observed owing to the high energy. The MW-assisted synthesis of CoFe_2_O_4_ nanocomposites (Fig. 11) displayed an interesting trend of morphological change patterns. As revealed in Fig. 11a and b, using MW as a powerful energy source afforded cubic nanostructures and non-uniform nanorod composites, respectively. The mean particle size of the cubic nanostructures was measured to be approximately 340 nm. In the case of the non-uniform nanorod morphology, the average diameter and the mean length were calculated to be 75 nm and 750 nm, respectively.

**Fig. 12:**
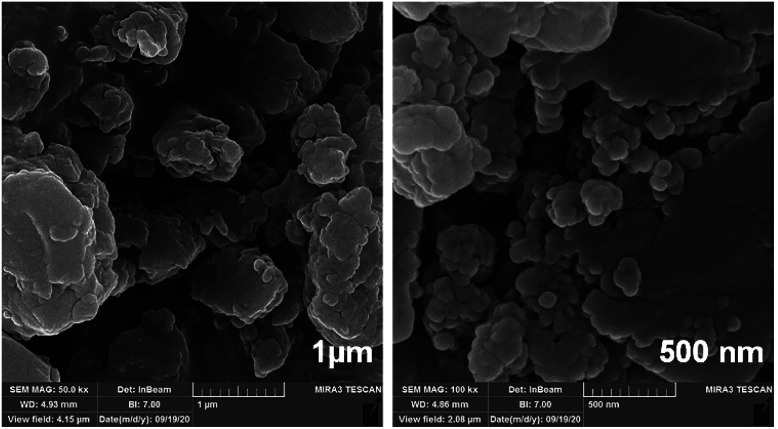
FE-SEM images of the N-GQDs/CoFe_2_O_4_ sphere nanocomposite.

**Fig. 13:**
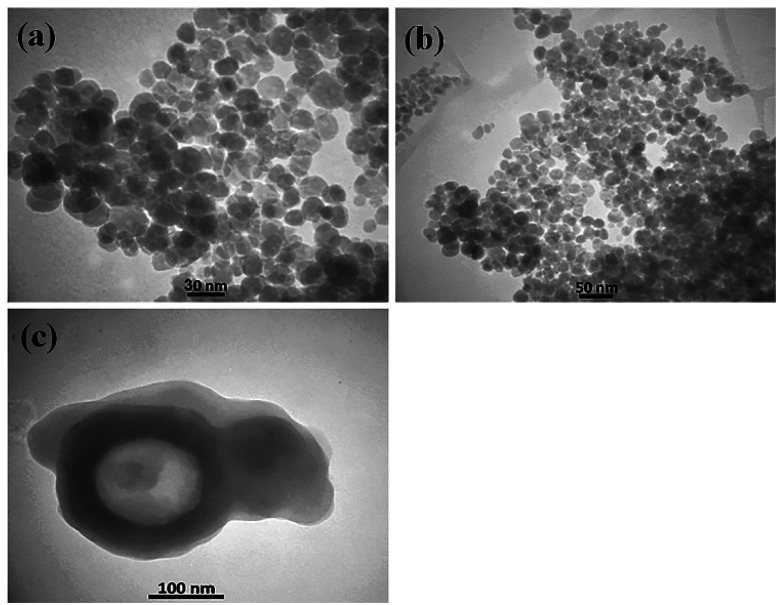
TEM images of the N-GQDs/CoFe_2_O_4_ sphere nanocomposite.

Because N-GQDs can accommodate an especially high amount of functional groups and edge surfaces, binding them to metal oxide nanocomposites results in stability of the catalyst during the reaction. Fig. 12 shows the FE-SEM images of the N-GQDs/CoFe_2_O_4_ nanocomposite. According to Fig. 12, the CoFe_2_O_4_ spherical morphology did not change after modification with N-GQDs. For further study of the morphology of the N-GQDs/CoFe_2_O_4_ nanocomposite, it was examined by a transmission electron microscope (TEM). As evidenced by the TEM images that appear in Fig. 13, the spherical morphology of N-GQDs/CoFe_2_O_4_ was formed from dense nanosphere building blocks through the self-assembly process.^51^

**Fig. 14:**
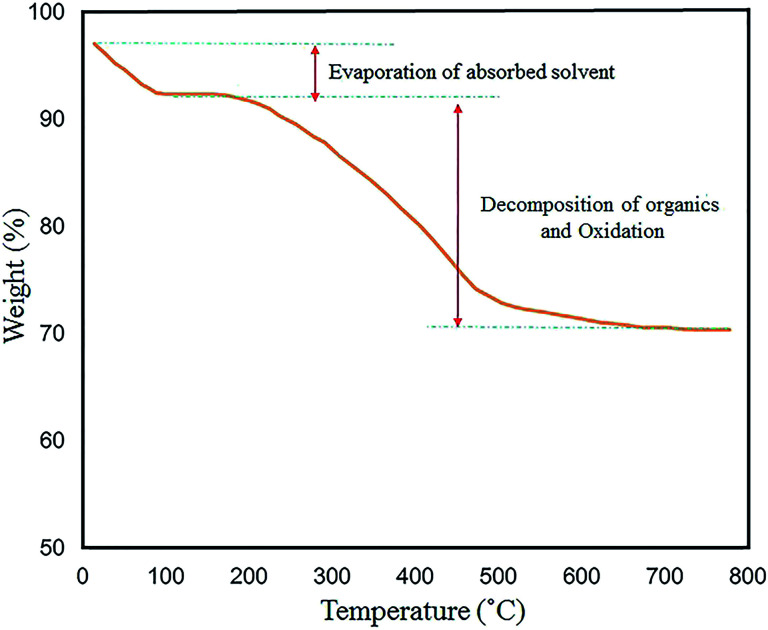
TG analysis of the N-GQDs/CoFe_2_O_4_ nanocomposites.

TG analysis was performed to examine the thermal stability of the N-GQDs/CoFe_2_O_4_ nanocomposite in the range of 30–800 °C. The thermal decomposition method is influenced by various conditions (*e.g.* heating rate, temperature, moisture content, and pressure). The total weight loss of 23.5% is displayed in Fig. 14. The first weight loss (∼5%) was observed at temperatures from 30 °C to 150 °C. This corresponds to the removal of the shell hydroxyl group and solvent bonding in physical adsorption. Also, the loss of approximately 20% from 200 °C to 550 °C is related to oxidation of the organic surface and decomposition of the N-GQDs/CoFe_2_O_4_ nanocomposite.

### 3.2. Catalytic study of the N-GQDs/CoFe_2_O_4_ sphere nanocomposite as a robust and retrievable catalyst in the microwave-assisted synthesis of quinoline-3-carbonitrile derivatives

In recent decades, many studies have been published on catalytic execution in the synthesis of quinoline-3-carbonitriles. Interestingly, each of these strategies has its competence for the synthesis of these compounds. Among them, organometallic nanocatalysts are being developed at a very quick pace. Hence, to compare the catalytic performance of the N-GQDs/CoFe_2_O_4_ nanocomposite with that of other reported catalysts for the synthesis of quinoline-3-carbonitriles,^52–56^ we have summarized the results in Table 2. In comparison with other mentioned results, the N-GQDs/CoFe_2_O_4_ nanocomposite has some advantages, including the highest yield of synthetic product and reasonable reaction times in mild conditions.

**Table 2:** Comparison of the N-GQDs/CoFe_2_O_4_ nanocomposite with some catalysts for the synthesis of 6-bromo-2-methoxy-4-(4-nitrophenyl)quinoline-3-carbonitrile

No.	Catalyst (amount)	Time	Yield^a^	TON^b^	Ref.
1	TiO_2_ nanopowder (100 mg)	70 s	85%	88	52
2	Dealuminated mesolite (150 mg)	30 min	93%	158	53
3	NH_4_OAc (155 mg)	6 h	80%	120	54
4	AlCl_3_ (100 mg)	7 h	88%	102	55
5	Nano-SBA-15 (20% mol)	4 h	96%	460	56
6	N-GQDs/CoFe_2_O_4_ (20 mg)	50 s	96%	280	This work

a Isolated yield.

b TON (turnover number): yield of product (wt. (mmol))/weight of the catalyst.

**Fig. 15:**
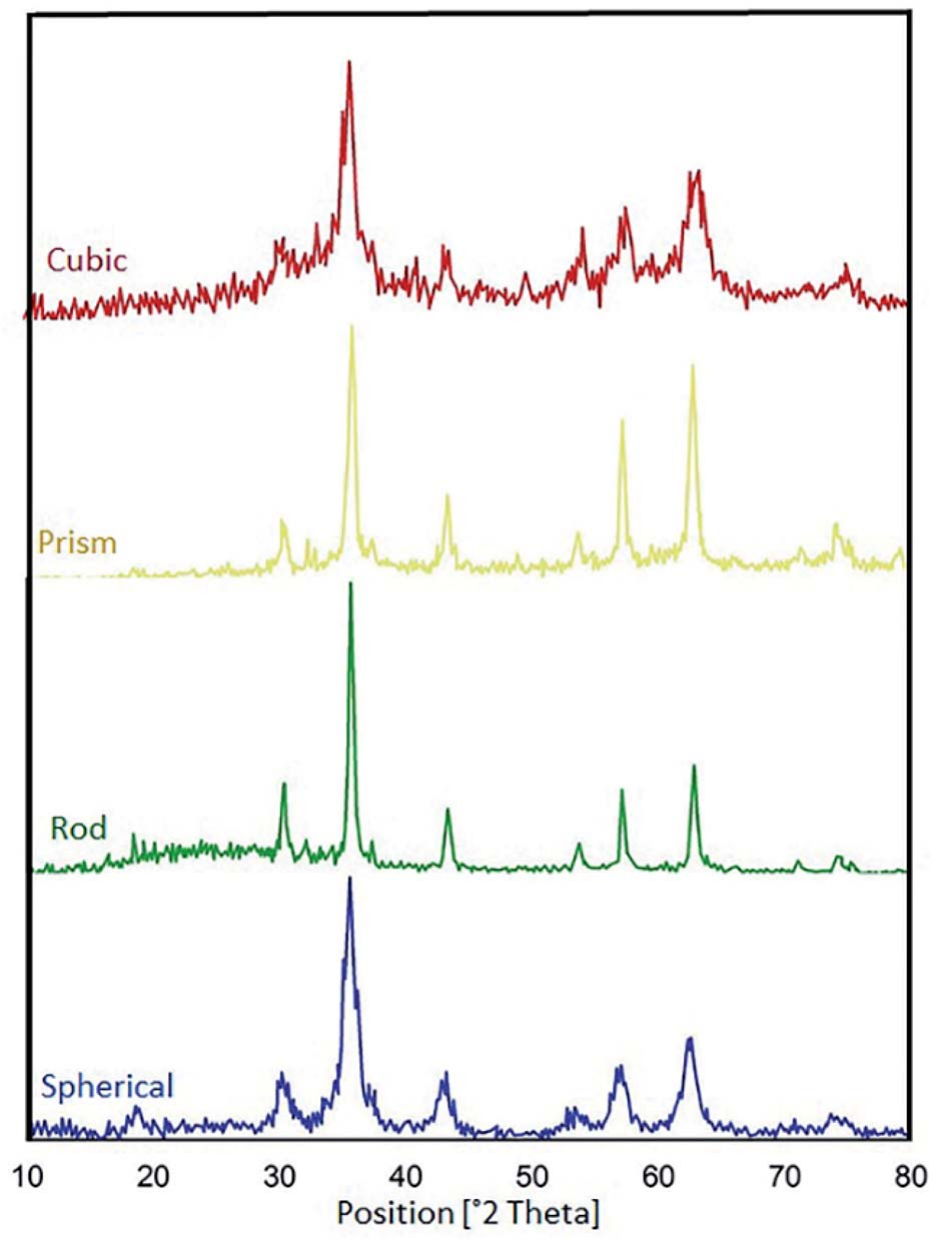
XRD graphs of the CoFe_2_O_4_ nanocomposites with various morphologies.

Based on the previous literature,^57,58^ the phase compositions of all the samples were investigated, and the XRD patterns of the CoFe_2_O_4_ nanocomposites with various morphologies are represented in Fig. 15. Also, Table 3 reveals data on the average crystalline sizes and particle sizes of the various morphologies of the CoFe_2_O_4_ nanocomposites. We consider the ratio *D*_FE-SEM_/*D*_XRD_ as a parameter that is indicative of the relationship between the regular and imperfect structural regions in the volume of the CoFe_2_O_4_ nanocomposites. In general, the catalytic activities of the various samples change when the *D*_FE-SEM_/*D*_XRD_ ratio is changed. We found that the best catalytic activities were observed for the spherical CoFe_2_O_4_ nanocomposite (*D*_FE-SEM_/*D*_XRD_ = 5.5).

**Table 3:** Particle sizes of the CoFe_2_O_4_ nanocomposites obtained from XRD and FE-SEM analysis

No.	Morphology	*D*_XRD_ a	*D*_FE-SEM_ b	*D*_FE-SEM_/*D*_XRD_
1	Spherical	10	55	5.5
2	Rod	2.9	75	25.86
3	Prism	2.1	80	38.09
4	Cubic	6.8	340	50

a *D*_XRD_: crystallite size was determined by Scherrer's equation.

b *D*_FE-SEM_: particle size was measured by FE-SEM analysis.

Microwave irradiation has been introduced as a green energy source in organic reactions. One of the great focuses of MW-assisted reactions is that it is easier and faster in comparison to conventional heating reactions.^59^ In this paper, our approach to create a superior and rapid route is the MW-assisted synthesis of quinoline-3-carbonitriles. The speed of the MW-assisted reaction and a comparison of the reaction times obtained with conventional heating reactions are shown in Table 4 (no. 1 and 2). Besides, the effects of morphology on the catalytic performance of the CoFe_2_O_4_ nanocomposites were tested in the MW-assisted one-pot multicomponent reaction of 4-nitrobenzaldehyde, methyl 2-cyanoacetate, and 4-bromoaniline as a model reaction (Table 4, no. 2–5). Among the various morphologies, the uniform spherical morphology obtained the best result. It seems that the spherical shape with high uniformity provides a more accessible surface and more active sites. Also, we found that the reaction does not progress well in the absence of a catalyst (Table 4, no. 6).

**Table 4:** Comparison of reaction times in both MW irradiation and classical thermal conditions in the preparation of quinoline-3-carbonitrile derivatives in the presence of various morphologies of the nanocatalyst^a^

No.	Product	Conditions	Time	Yield^b^ (%)
1	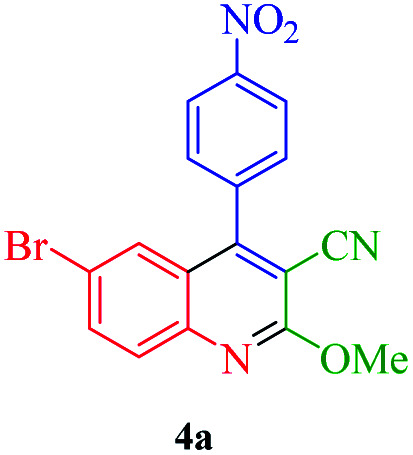	Δ/nano-CoFe_2_O_4_ sphere	15 min	75
2		MW/nano-CoFe_2_O_4_ sphere	50 s	87
3		MW/nano-CoFe_2_O_4_ prism	50 s	84
4		MW/nano-CoFe_2_O_4_ cubic	50 s	82
5		MW/nano-CoFe_2_O_4_ rod	50 s	85
6		Δ/no cat.	180 min	<5

a Reaction conditions: 4-nitrobenzaldehyde (1 mmol), methyl 2-cyanoacetate (1 mmol), and 4-bromoaniline (1 mmol).

b Isolated yield.

In further continuation of this study, the catalytic execution of the N-GQDs/CoFe_2_O_4_ nanocomposite as a robust and retrievable nanocatalyst was explored in a one-pot reaction of quinoline-3-carbonitriles under green and mild conditions. Initially, systematic research was conducted to achieve optimum conditions (solvent and amount of catalyst). Therefore, we examined the three-component reaction of 4-nitrobenzaldehyde, methyl 2-cyanoacetate, and 4-bromoaniline as a model reaction. The collected optimized results are shown in Table 5. The effects of various solvents (*e.g.* chloroform, acetonitrile, water, and ethanol) were employed in the model reaction. As is also shown, the protic solvents provided satisfactory results. As compared with H_2_O (78% yield), the reaction operated well in ethanol (96% yield). According to the obtained data, ethanol was selected as a green and suitable solvent for the synthesis of quinoline-3-carbonitriles. Next, to investigate the effects of diverse catalysts, Co_3_O_4_, Fe_3_O_4_, CoFe_2_O_4_, and N-GQDs/CoFe_2_O_4_ nanocomposites were tested, and we found that the reaction gave convincing results in the presence of the N-GQDs/CoFe_2_O_4_ sphere nanocomposite. Finally, the amount of nanocatalyst was explored under optimum conditions. When the quantity was changed to 10, 15, and 20 mg, the reaction yield was increased (91–96%). However, no change in the reaction yield was observed with increasing nanocatalyst amount (from 20 mg to 25 mg).

**Table 5:** Optimization of the conditions of the model reaction^a^

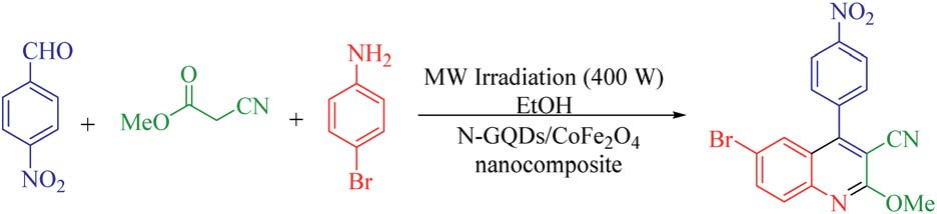				
No.	Solvent	Catalyst amount	Time (s)	Yield^c^ (%)
1	CHCl_3_	20 mg	90	40
2	MeCN	20 mg	80	67
3	H_2_O	20 mg	100	78
4	EtOH	Nano-Co_3_O_4_ (30 mg)	105	51
5	EtOH	Nano-Fe_3_O_4_ (30 mg)	95	62
6	EtOH	Nano-CoFe_2_O_4_ – (25 mg)	85	87
7	EtOH	10 mg	70	91
8	EtOH	15 mg	60	94
**9**	**EtOH**	**20 mg**	**50**	**96** b
10	EtOH	25 mg	50	96

a One-pot of methyl 2-cyanoacetate (1 mmol), 4-nitrobenzaldehyde (1 mmol), and 4-bromoaniline (1 mmol) under MW conditions.

b The bold values reveals the best conditions for the reaction.

c Isolated yield.

According to the optimized conditions, we studied the MW-assisted reaction of methyl 2-cyanoacetate with various substrates of aryl aldehydes and aromatic amines. From Table 6, it is clear that aryl aldehydes with electron-withdrawing groups react faster in comparison to those with electron-releasing groups. This may well be owing to the electron-withdrawing groups of various substituted aryl aldehydes and the faster nucleophilic attack of methyl 2-cyanoacetate.

**Table 6:** Synthesis of quinoline-3-carbonitrile derivatives in the presence of the N-GQDs/CoFe_2_O_4_ nanocomposite as a catalyst under MW irradiation conditions

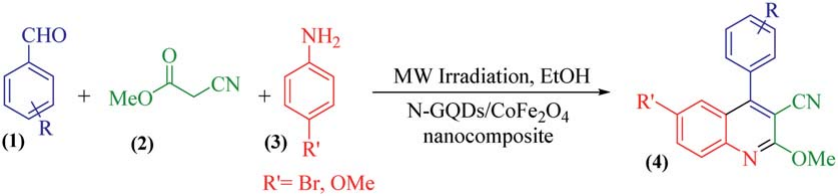
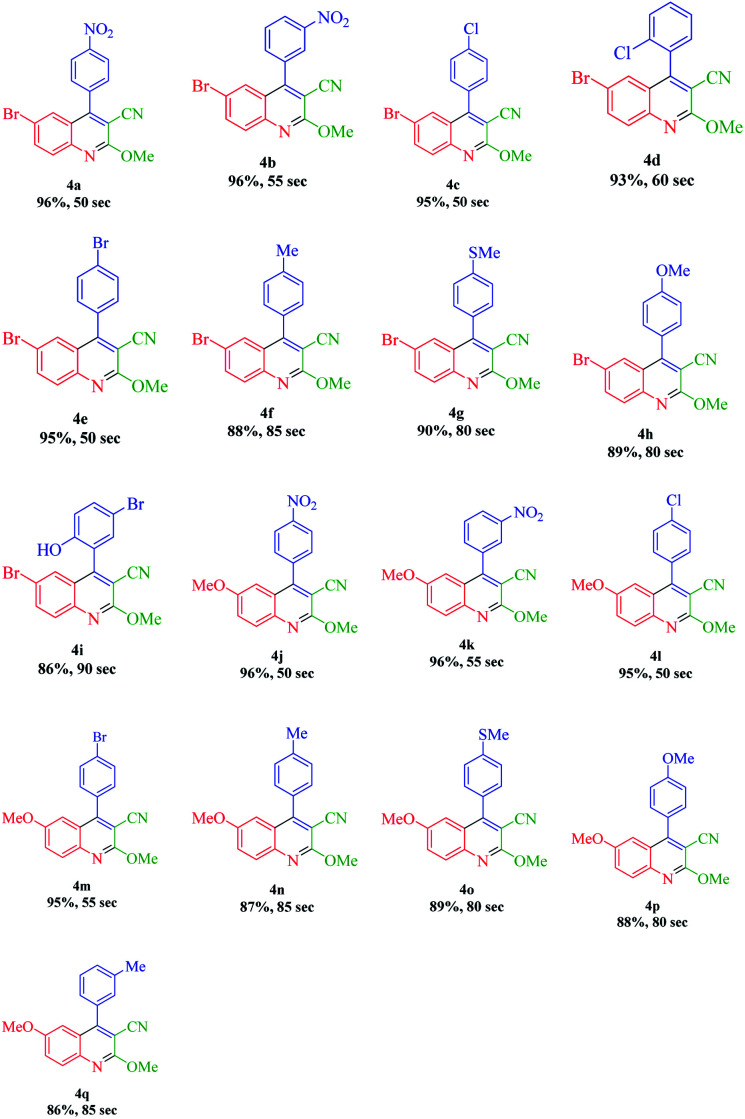

**Scheme 3:**
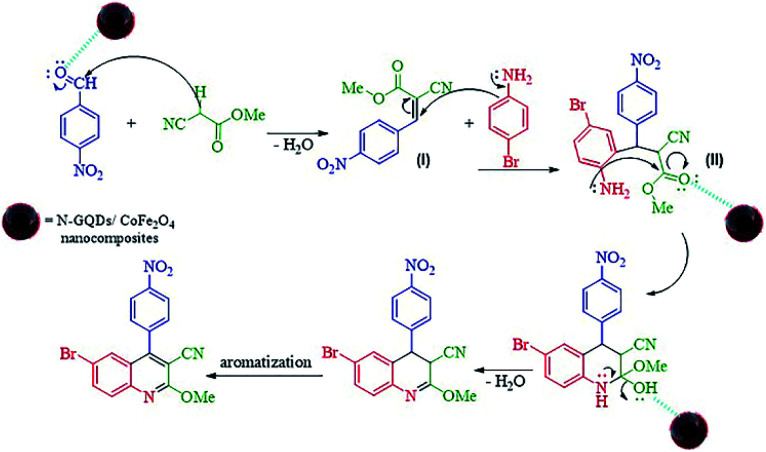
Proposed mechanism for the formation of 4ba.

The probable reaction mechanism for the MW-assisted synthesis of 6-bromo-2-methoxy-4-(4-nitrophenyl)quinoline-3-carbonitrile in the presence of N-GQDs/CoFe_2_O_4_ sphere nanostructures from the three-component reaction of methyl 2-cyanoacetate, 4-nitrobenzaldehyde, and 4-bromoaniline is demonstrated in Scheme 3.

It seems that the functional groups on the surface and the edges of the N-GQDs/CoFe_2_O_4_ nanocomposites play the main role in the catalytic activity. In other words, the electrophilic character of the aryl aldehyde was increased by the catalytic activity of the N-GQDs shell, including the H-bonds between the functional groups on the N-GQDs and O atom of the carbonyl group. Therefore, the nucleophilic attack of methyl 2-cyanoacetate on the aryl aldehyde is enhanced by N-GQDs/CoFe_2_O_4_ as a nanocatalyst. Intermediate (I) was formed from the condensation of methyl 2-cyanoacetate and 4-nitrobenzaldehyde *via* Knoevenagel condensation. Afterward, intermediate (II) was formed from the Michael addition of 4-bromoaniline to intermediate (I). Then, intramolecular cyclization was observed in the next step. Then, intramolecular cyclization with the elimination of water was observed in the next step. The desired product was finally formed by aromatization.

### 3.3. Reusability of the nanocatalyst

**Fig. 16:**
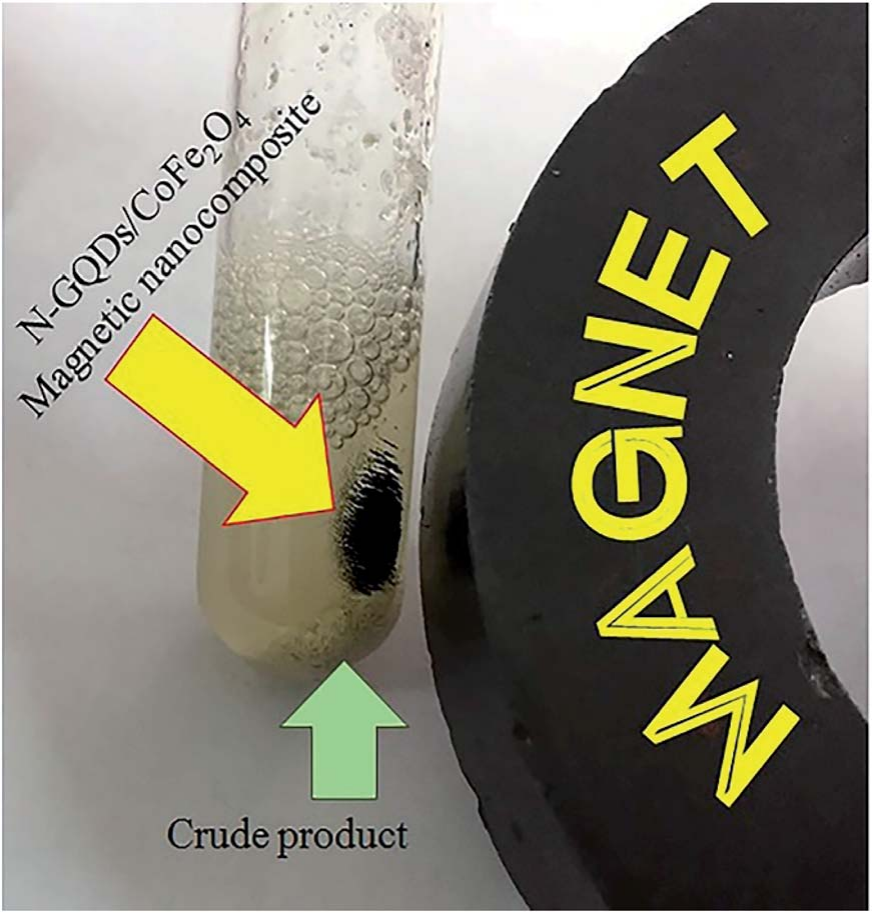
Digital camera image of the N-GQDs/CoFe_2_O_4_ magnetic nanocomposites after application of an external magnet.

**Fig. 17:**
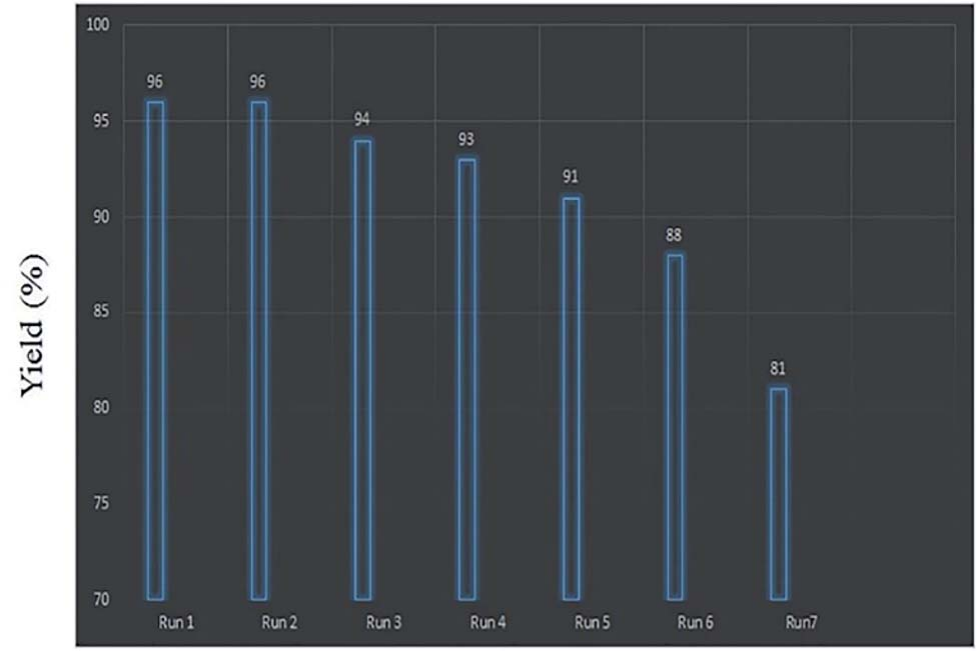
Retrievability of the nanocatalyst.

**Fig. 18:**
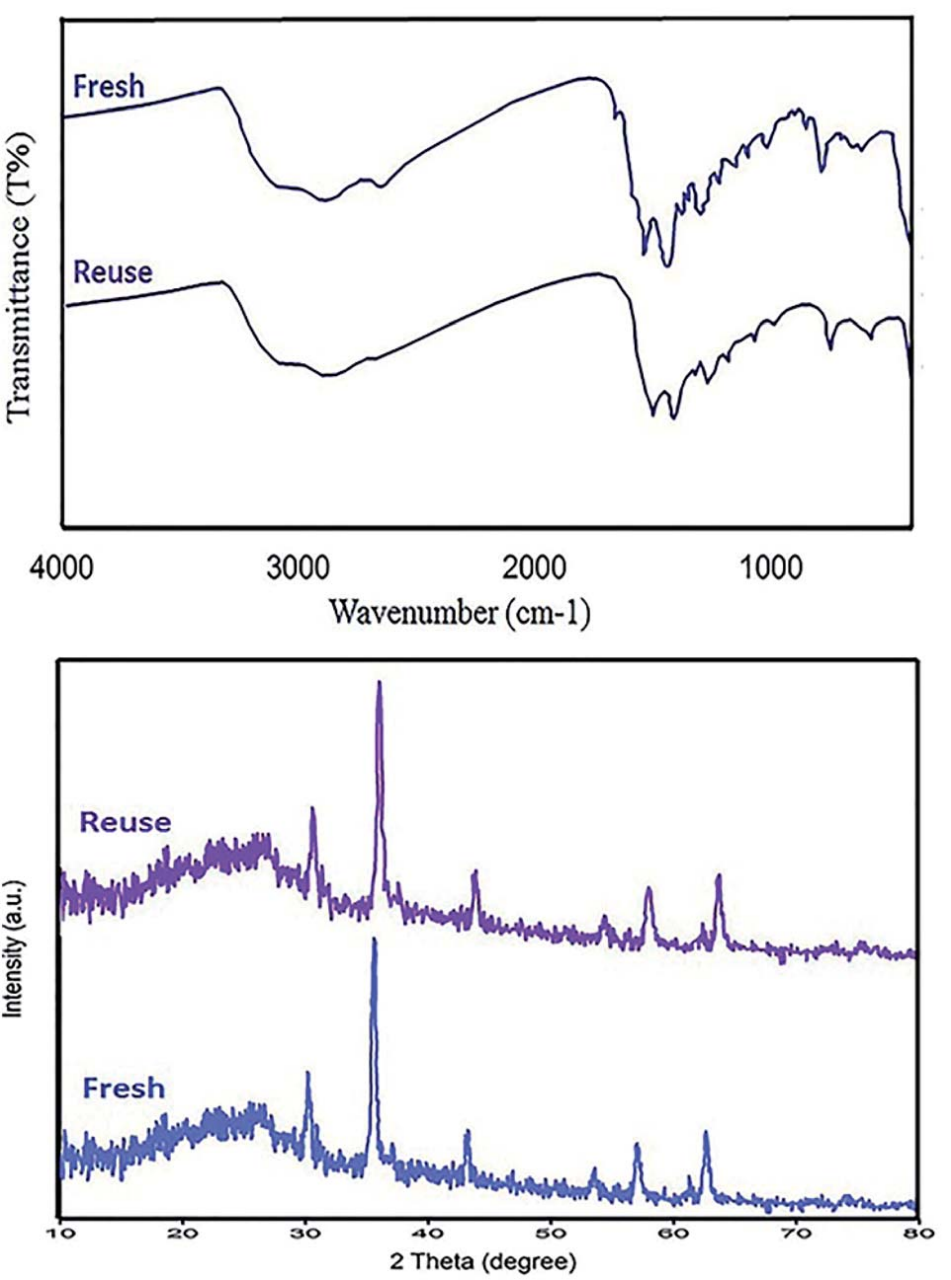
FT-IR spectrum (top) and XRD pattern (bottom) of the recovered N-doped GQDs/CoFe_2_O_4_ nanocomposite after 7 runs.

Retrievability is one of the main factors associated with the use of nanocatalysts. Based on the magnetic properties of the nanocatalyst, after the completion of the reaction, the nanocatalyst could be separated by an external magnet from the reaction mixture (see Fig. 16). Then, it was washed with dry EtOH several times and dried at 60 °C overnight. It was found that for each run, the corresponding product was obtained in excellent yield and selectivity, although with a longer reaction time and a slight decrease in yield compared to the first run, indicating high reactivity, durability, and stability of the catalyst (Fig. 17). Also, the acid sites (1.23 mmol g^−1^) of the catalyst after being reused 7 times had no dramatic changes based on the acid–base titration measurement, in comparison with the acid sites of the fresh N-doped GQDs/CoFe_2_O_4_ nanocomposite (1.26 mmol g^−1^); these facts confirm that the efficiency, appearance, and structure of the N-doped GQDs/CoFe_2_O_4_ nanocomposite remained intact in the recycles, and there was no considerable deformation or leaching after 7 runs. Moreover, the chemical structure of the recovered N-doped GQDs/CoFe_2_O_4_ nanocomposite was confirmed by FT-IR and XRD analyses. There is no significant difference between the XRD and FT-IR spectra of the fresh and recovered nanocomposites. Fig. 18 depicts the FT-IR and XRD patterns of the N-GQDs/CoFe_2_O_4_ nanocomposites after 7 runs of reuse.

## 4. Conclusion

In this paper, the morphology-dependent catalytic performance of CoFe_2_O_4_ nanocomposites was systematically investigated. Diverse morphologies, such as nanoprism, nanorod, nanocubic, and nanosphere CoFe_2_O_4_ composites, were successfully prepared *via* MW and hydrothermal routes in various conditions for the first time. When utilized as a nanocatalyst for the synthesis of 6-bromo-2-methoxy-4-(4-nitrophenyl)quinoline-3-carbonitrile, the uniform spherical CoFe_2_O_4_ nanocomposite presented superior catalytic activity. The remarkable catalytic activity was greatly related to the uniformity and spherical shape with available active sites. Then, the CoFe_2_O_4_ sphere composites were decorated with N-GQDs. It was found that the joining of N-GQDs and CoFe_2_O_4_ has a remarkable impact on the catalytic activity improvement. The as-fabricated N-GQDs/CoFe_2_O_4_ nanocomposite displayed considerable attributes, including high thermal stability, great particle uniformity, chemical stability, and excellent catalytic activity. Consequently, a green MW-assisted route was achieved for one-pot synthesis of new and known quinoline-3-carbonitrile derivatives in the presence of the N-GQDs/CoFe_2_O_4_ nanocomposite in the shortest appropriate time. The examination of the morphology-dependent catalytic performance of CoFe_2_O_4_ and using N-GQDs as a modified layer will provide a new-fashioned perspective for the quick advancement of CoFe_2_O_4_-based catalysts.

## Author contributions

These authors contributed to the design of the research, analysis of the results, and writing of the manuscript equally.

## Conflicts of interest

There are no conflicts to declare.

## Supplementary Material

RA-011-D1RA05739A-s001
