# Supplementary material for: Engineered N-doped graphene quantum dots/CoFe2O4 spherical composites as a robust and retrievable catalyst: fabrication, characterization, and catalytic performance investigation in microwave-assisted synthesis of quinoline-3-carbonitrile derivatives
Source: RSC Adv. 2021 Oct 27;11(55):34724–34. doi: 10.1039/d1ra05739a (PMC9042697; doi:10.1039/d1ra05739a)
Supplement: RA-011-D1RA05739A-s001 [file RA-011-D1RA05739A-s001.pdf]

Electronic Supplementary Material (ESI) for RSC Advances.
This journal is © The Royal Society of Chemistry 2021

Engineered N-GQDs/$CoFe_2O_4$ Spherical Composites as A Robust and Retrievable Catalyst: Fabrication, Characterization, and Investigation Catalytic Performance in Microwave-assisted synthesis of quinoline-3-carbonitrile derivatives

Pouria Babaei and Javad Safaei-Ghomi*

[a]*Department of Organic Chemistry, Faculty of Chemistry, University of Kashan, Kashan, 51167, I. R. Iran*

General procedure for the Synthesis of quinoline-3-carbonitrile derivatives

A 25 ml round bottom flask was charged with the same molar ratio of various aryl aldehydes (**1**), methyl cyanoacetate (**2**), different substituted aniline (**3**), N-GQDs/$CoFe_2O_4$ (20 mg), and ethanol. The whole mixture was stirred for 1 minute at ambient temperature. After well mixing, the prepared mixture was exposed to microwave irradiation at 400 W. Upon completion of the time reaction (monitored by TLC), the crude product was filtered and washed with cold dry ethanol to get pure product.

Physical and Specteral data

6-bromo-2-methoxy-4-(4-nitrophenyl)quinoline-3-carbonitrile (4a): White solid, m.p. 129-132 °C; FT-IR (KBr) (ν_{max}/cm^{-1}): 3010, 2951, 2213, 1730, 1585, 1090; ^1H NMR (400 MHz, DMSO-*d₆*): δ (ppm) 3.98 (3H, s, OMe), 7.26 (1H, s, CH Ar,), 8.11- 8.15 (2H, d, *J*= 16 Hz, CH Ar,), 8.31 (2H, bs, CH Ar), 8.33- 8.37 (2H, d, *J*= 16 Hz, CH Ar).

6-bromo-2-methoxy-4-(3-nitrophenyl)quinoline-3-carbonitrile (4b): White solid, m.p. 129-131 °C; FT-IR (KBr) (ν_{max}/cm^{-1}): 3012, 2945, 2218, 1738, 1589, 1081; ^1H NMR (400 MHz, DMSO-d_6): δ (ppm) 3.97 (3H, s, OMe), 7.26 (1H, s, CH Ar), 7.71-7.77 (1H, t, *J*= 12 Hz, CH Ar), 8.32 (2H, bs, CH Ar), 8.39- 8.42 (2H, d, *J*= 12 Hz, CH Ar), 8.70 (1H, s, CH Ar).

6-bromo-2-methoxy-4-(4-chlorophenyl)quinoline-3-carbonitrile (4c): White solid, m.p. 134-132 °C; FT-IR (KBr) (ν_{max}/cm^{-1}): 3022, 2951, 2220, 1731, 1581, 1078; ^{1}H NMR (400 MHz, DMSO-d_6): δ (ppm) 3.94 (3H, s, OMe), 7.26 (2H, s, CH Ar), 7.47- 7.50 (2H, CH Ar, d, J= 12 Hz), 7.92-7.95 (2H, CH Ar, d, J= 12 Hz), 8.21 (1H, s, CH Ar).

6-bromo-2-methoxy-4-(2-chlorophenyl)quinoline-3-carbonitrile (4d): White solid, m.p. 128-130 °C; FT-IR (KBr) (ν_{max}/cm^{-1}): 3019, 2954, 2218, 1728, 1580, 1065; ^{1}H NMR (400 MHz, DMSO-d_6): δ (ppm) 3.96 (3H, s, OMe), 7.26- 7.50 (3H, m, CH Ar), 7.78- 8.17 (1H, s CH Ar), 8.22- 8.25 (2H, d, J= 12 Hz, CH Ar), 8.70 (1H, s, CH Ar).

6-bromo-2-methoxy-4-(4-bromophenyl)quinoline-3-carbonitrile (4e): White solid, m.p. 125-126 °C; FT-IR (KBr) (ν_{max}/cm^{-1}): 3028, 2933, 2228, 1718, 1585, 1068; ^{1}H NMR (400 MHz, DMSO-d_6): δ (ppm) 3.94 (3H, s, OMe), 7.26 (2H, bs, CH Ar), 7.47- 7.50 (2H, d, J= 12 Hz, CH Ar), 7.92-7.95 (2H, d, J= 12 Hz, CH Ar), 8.21 (1H, s, CH Ar).

6-bromo-2-methoxy-4-(4-methylphenyl)quinoline-3-carbonitrile (4f): White solid, m.p. 133-135 °C; FT-IR (KBr) (ν_{max}/cm^{-1}): 3018, 2918, 2231, 1728, 1575, 1070; ^{1}H NMR (400 MHz, DMSO-d_6): δ (ppm) 2.43 (3H, s, CH_3), 3.93 (3H, s, OMe), 7.26 (1H, s, CH Ar), 7.29- 7.32 (2H, d, J=12 Hz, CH Ar,), 7.62 (1H, s, CH Ar), 7.89- 7.92 (2H, d, J=12 Hz, CH Ar), 8.23 (1H, s, CH Ar).

6-bromo-2-methoxy-4-(4-(methylthio)phenyl)quinoline-3-carbonitrile (4g): Light yellow solid, m.p. 130-132 °C; FT-IR (KBr) (ν_{max}/cm^{-1}): 3018, 2947, 2218, 1728, 1589, 1088; ^{1}H NMR (400 MHz, DMSO-d_6): δ (ppm) 2.53 (3H, s, SMe), 3.92 (3H, s, OMe), 7.28- 7.31 (2H, d, J= 12 Hz, CH Ar,), 7.63 (2H, s, CH Ar), 7.90- 7.93 (2H, d, J= 12 Hz, CH Ar), 8.18 (1H, s, CH Ar); ^{13}C NMR (100 MHz, DMSO-d_6): δ (ppm) 14.61, 53.28, 99.83, 100.44, 115.86, 119.89, 125.43, 126.60, 127.05, 127.15, 127.31, 127.53, 131.46, 138.30, 143.31, 149.80, 154.46, 60.22.

6-bromo-2-methoxy-4-(4-methoxyphenyl)quinoline-3-carbonitrile (4h): White solid, m.p. 128-131 °C; FT-IR (KBr) (ν_{max}/cm^{-1}): 3010, 2957, 2214, 1720, 1585, 1092; ^{1}H NMR (400 MHz, DMSO-d_6): δ (ppm) 3.89 (3H, s, OMe), 3.91 (3H, s, OMe), 6.67- 7.01 (2H, d, *J*= 12 Hz, CH Ar), 7.62 (2H, s, CH Ar), 7.98- 8.02 (2H, d, *J*= 12 Hz, CH Ar), 8.18 (1H, s, CH Ar).

6-bromo-4-(5-bromo-2-hydroxyphenyl)-2-methoxyquinoline-3-carbonitrile (4i): Yellow solid, m.p. 134-136 °C; FT-IR (KBr) (ν_{max}/cm^{-1}): 3010, 2957, 2214, 1720, 1585, 1092; ^{1}H NMR (400 MHz, DMSO-d_6): δ (ppm) 3.81 (3H, s, OMe), 6.69 (1H, bs, OH), 7.40- 7.51 (3H, m, CH Ar), 7.55-7.62 (3H, m, CH Ar).

2,6-dimethoxy-4-(4-nitrophenyl)quinoline-3-carbonitrile (4j): White solid, m.p. 133-135 °C; FT-IR (KBr) (ν_{max}/cm^{-1}): 3010, 2957, 2214, 1720, 1585, 1092; ^{1}H NMR (400 MHz, DMSO-d_6): δ (ppm) 3.89 (3H, s, CH OMe), 3.98 (3H, s, CH OMe), 7.98 (1H, m, CH Ar), 8.11- 8.15 (2H, d, *J*= 16 Hz, CH Ar), 8.31 (1H, CH Ar), 8.33- 8.37 (2H, d, *J*= 16 Hz, CH Ar).

2,6-dimethoxy-4-(3-nitrophenyl)quinoline-3-carbonitrile (4k): White solid, m.p. 133-135 °C; FT-IR (KBr) (ν_{max}/cm^{-1}): 3012, 2958, 2218, 1718, 1580, 1099; ^{1}H NMR (400 MHz, DMSO-d_6): δ (ppm) 3.97 (6H, s, OMe), 7.70- 7.76 (2H, t, *J*= 12 Hz, CH Ar), 8.31 (2H, bs, CH Ar), 8.39- 8.42 (3H, d, *J*= 12 Hz, CH Ar), 8.70 (1H, s, CH Ar).

2,6-dimethoxy-4-(4-chlorophenyl)quinoline-3-carbonitrile (4l): White solid, m.p. 130-132 °C; FT-IR (KBr) (ν_{max}/cm^{-1}): 3018, 2968, 2228, 1719, 1580, 1091; ^{1}H NMR (400 MHz, DMSO-d_6): δ (ppm) 3.93 (6H, s, OMe), 7.25 (1H, s, CH Ar), 7.46- 7.49 (2H, d, *J*= 12 Hz, CH Ar), 7.92- 7.95 (2H, d, *J*= 12.4 Hz, CH Ar), 8.20 (2H, bs, CH Ar).

2,6-dimethoxy-4-(4-bromophenyl)quinoline-3-carbonitrile (4m): White solid, m.p. 130-132 °C; FT-IR (KBr) (ν_{max}/cm^{-1}): 3020, 2966, 2235, 1721, 1576, 1085; ^{1}H NMR (400 MHz, DMSO-d_6): δ

(ppm) 3.89 (3H, s, OMe), 3.91 (3H, s, OMe), 6.98- 7.01 (2H, d, J= 12Hz, CH Ar), 7.26 (2H, bs, CH Ar), 7.99- 7.02 (2H, d, *J*= 12 Hz, CH Ar), 8.18 (1H, s, CH Ar).

2,6-dimethoxy-4-(4-methylphenyl)quinoline-3-carbonitrile (4n): White solid, m.p. 130-132 °C; FT-IR (KBr) (ν_{max}/cm^{-1}): 3033, 2981, 2215, 1720, 1567, 1095; ^{1}H NMR (400 MHz, DMSO-d_6): δ (ppm) 1.55 (3H, s, Me), 3.94 (3H, s, OMe), 4.37 (3H, s, OMe), 7.20- 7.26 (3H, m, CH Ar), 8.01- 8.06 (2H, m, CH Ar), 8.22 (2H, bs, CH Ar).

2,6-dimethoxy-4-(4-(methylthiophenyl)quinoline-3-carbonitrile (4o): light Yellow solid, m.p. 133-135 °C; FT-IR (KBr) (ν_{max}/cm^{-1}): 3033, 2986, 2230, 1712, 1575, 1089; ^{1}H NMR (400 MHz, DMSO-d_6): δ (ppm) 2.53 (3H, s, CH SMe), 3.89 (3H, s, OMe), 3.92 (3H, s, OMe), 7.10 (2H, bs, CH Ar), 7.27- 7.30 (2H, d, *J*= 12 Hz, CH Ar), 7.89- 7.92 (2H, d, *J*= 12 Hz, CH Ar), 8.17 (1H, s, CH Ar); ^{13}C NMR (100 MHz, DMSO-d_6): δ (ppm) 14.61, 53.28, 53.49, 91.39, 100.45, 112.94, 113.58, 115.89, 116.06, 125.42, 127.52, 128.76, 128.98, 131.46, 147.44, 154.46, 155.81, 161.63, 179.79.

2,6-dimethoxy-4-(4-methoxyphenyl)quinoline-3-carbonitrile (4p): White solid, m.p. 132-133 °C; FT-IR (KBr) (ν_{max}/cm^{-1}): 3034, 2988, 2231, 1715, 1579, 1092; ^{1}H NMR (400 MHz, DMSO-d_6): δ (ppm) 3.89 (6H, s, OMe), 3.91 (3H, s, OMe), 6.98- 7.01 (2H, d, *J*= 12 Hz, CH Ar), 7.26 (1H, s, CH Ar), 7.99- 8.02 (2H, d, *J*= 12 Hz, CH Ar), 8.18 (2H, bs, CH Ar).

2,6-dimethoxy-4-(methyl)quinoline-3-carbonitrile (4q): Light Yellow solid, m.p. 129-131 °C; FT-IR (KBr) (ν_{max}/cm^{-1}): 3028, 2980, 2218, 1710, 1573, 1078; ^{1}H NMR (400 MHz, DMSO-d_6): δ (ppm) 1.55 (3H, s, Me), 2.43 (3H, s, OMe), 3.92 (3H, s, OMe), 7.25 (1H, s, CH Ar), 7.29- 7.32 (3H, m, CH Ar), 7.88- 8.91 (2H, d, *J*= 12 Hz, CH Ar), 8.22 (1H, s, CH Ar).

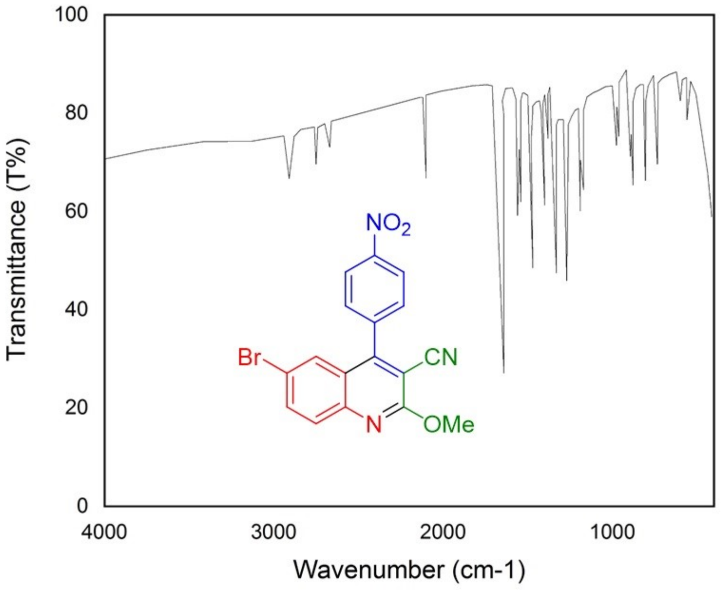

Figure S1. FT-IR spectrum of compound 4a

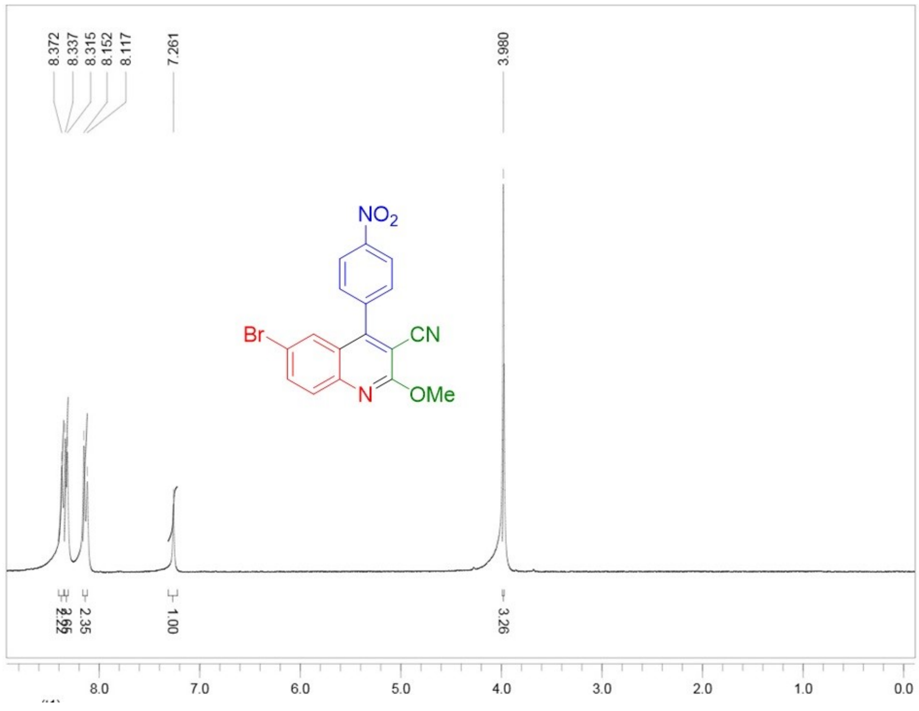

Figure S2. ^{1}H NMR spectrum of compound 4a

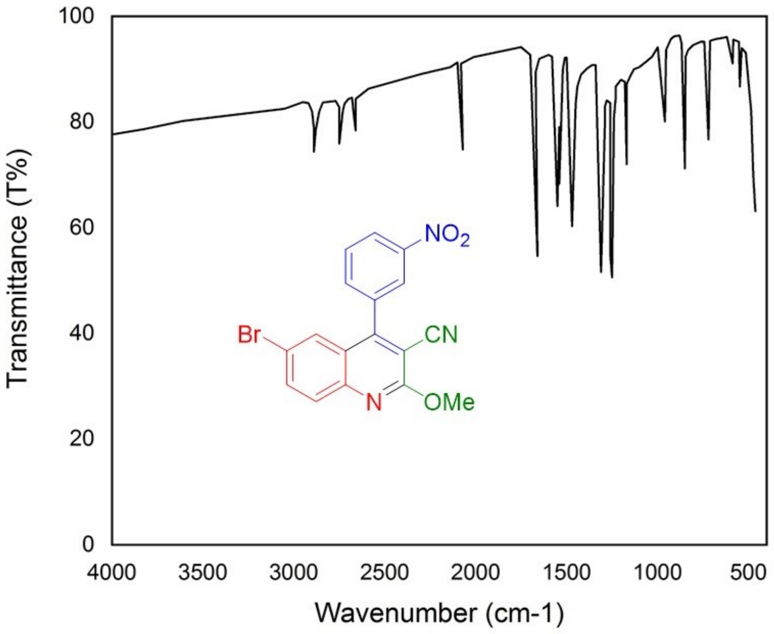

Figure S3. FT-IR spectrum of compound 4b

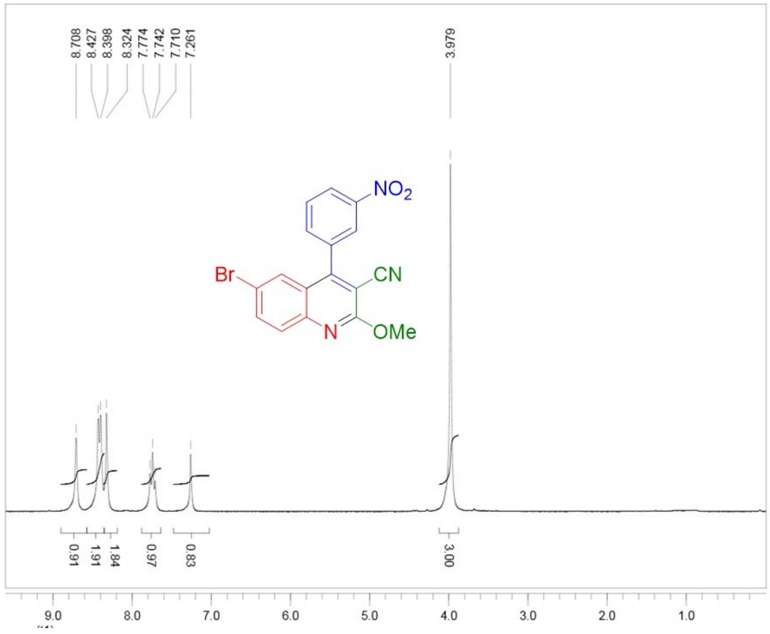

Figure S4. ^{1}H NMR spectrum of compound 4b

Figure S5. FT-IR spectrum of compound 4c

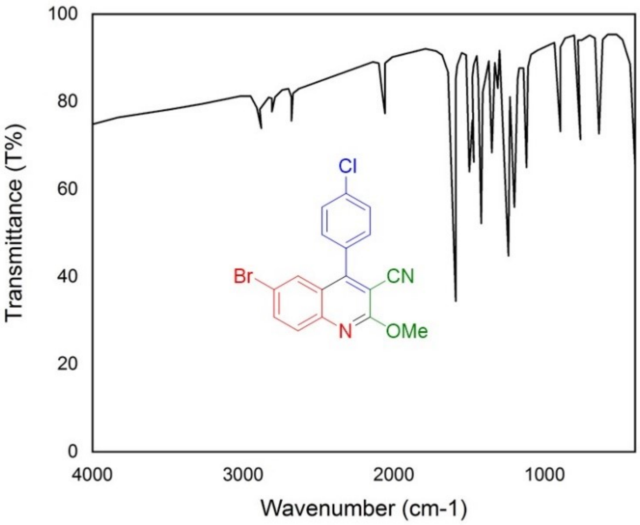

Figure S6. ^{1}H NMR spectrum of compound 4c

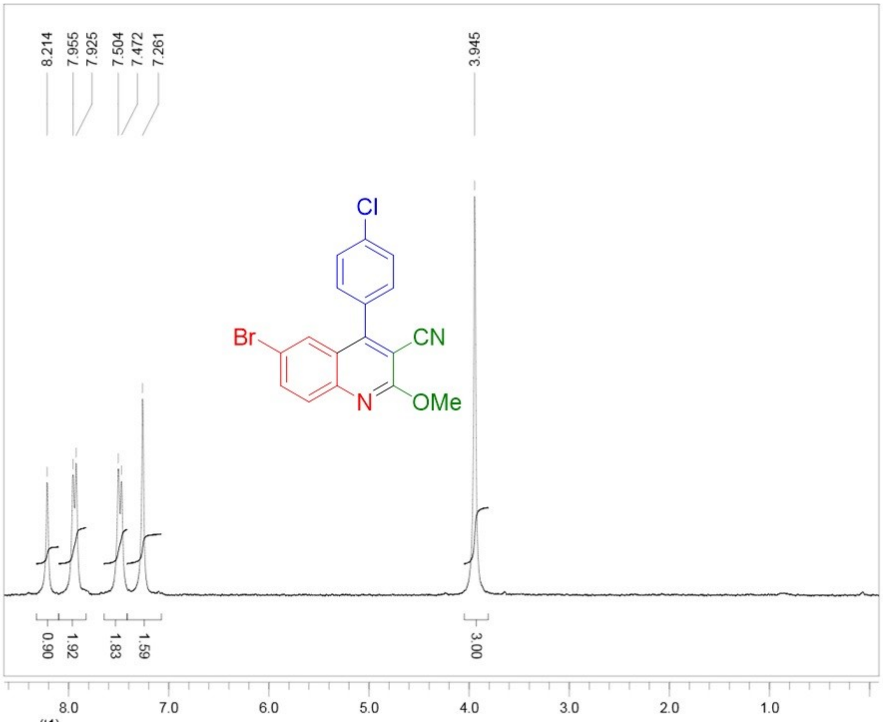

Figure S7. FT-IR spectrum of compound 4d

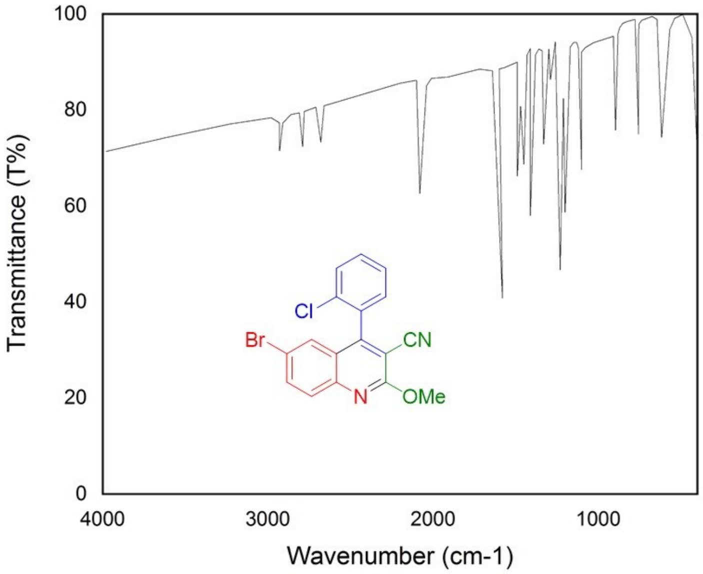

Figure S8. ^{1}H NMR spectrum of compound 4d

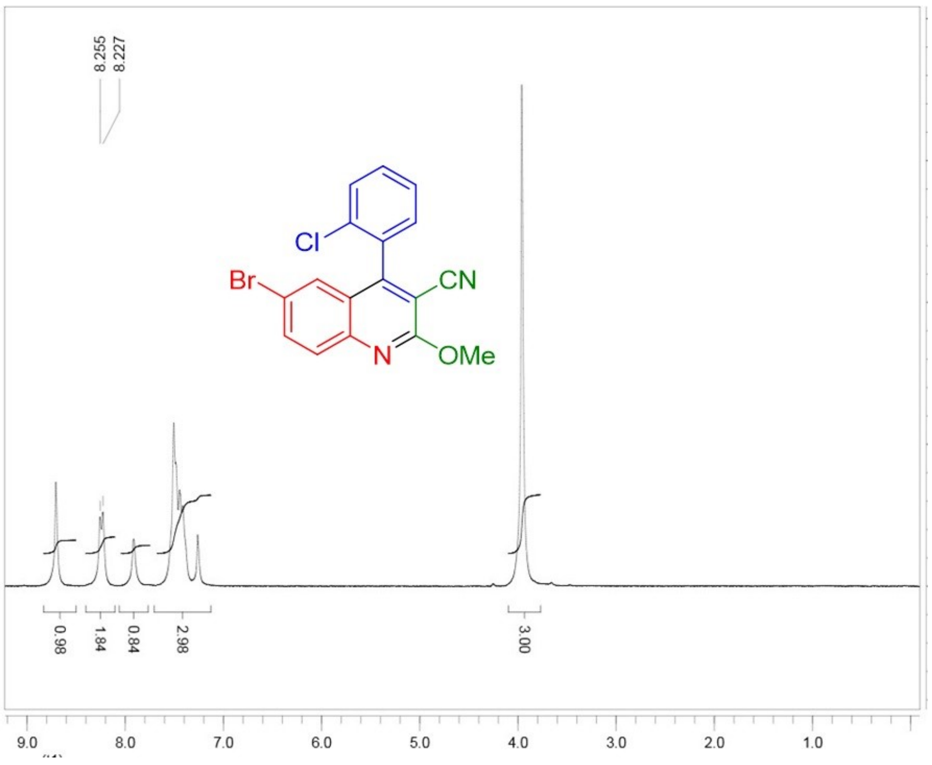

Figure S9. FT-IR spectrum of compound 4e

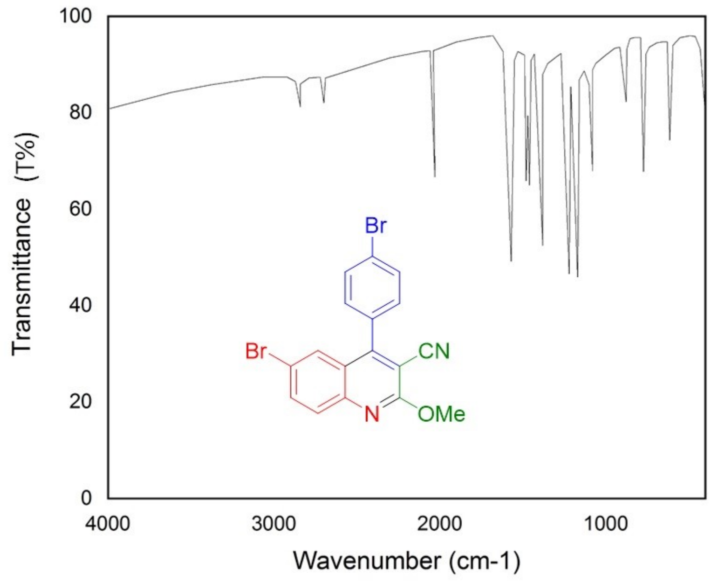

Figure S10. ^{1}H NMR spectrum of compound 4e

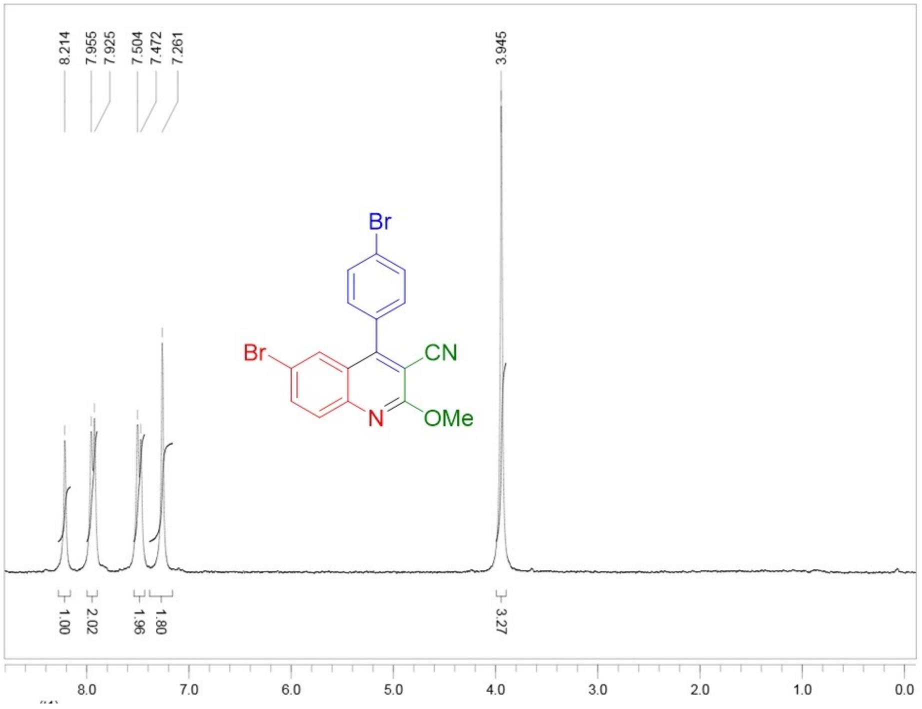

Figure S11. FT-IR spectrum of compound 4f

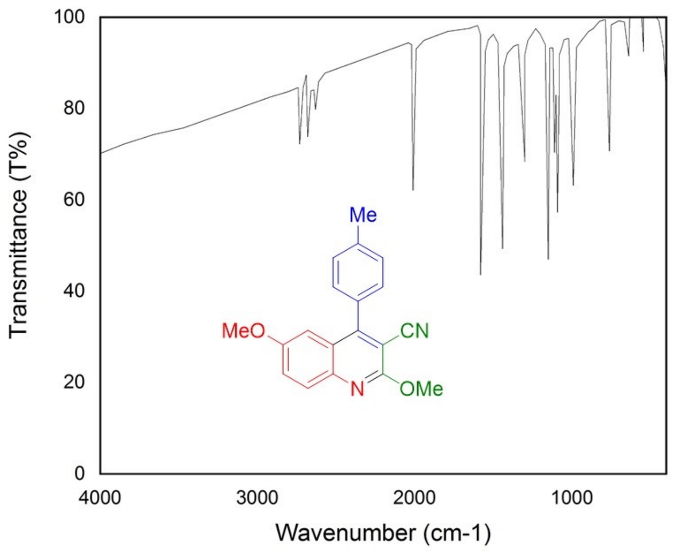

Figure S12. ^{1}H NMR spectrum of compound 4f

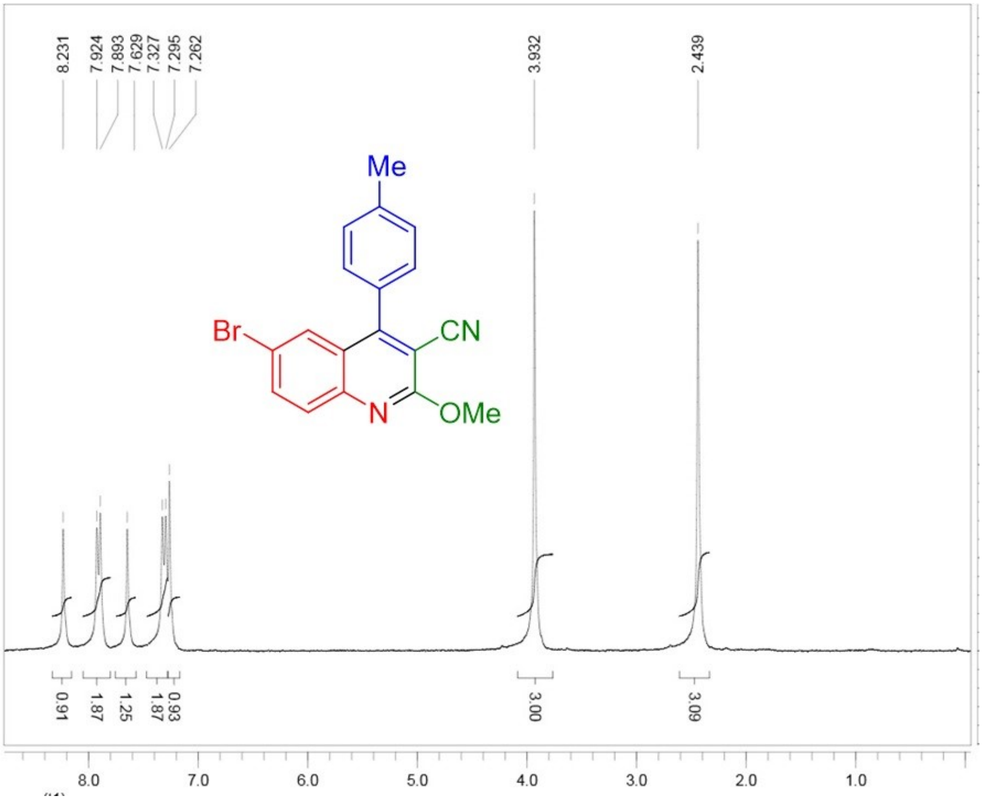

Figure S13. FT-IR spectrum of compound 4g

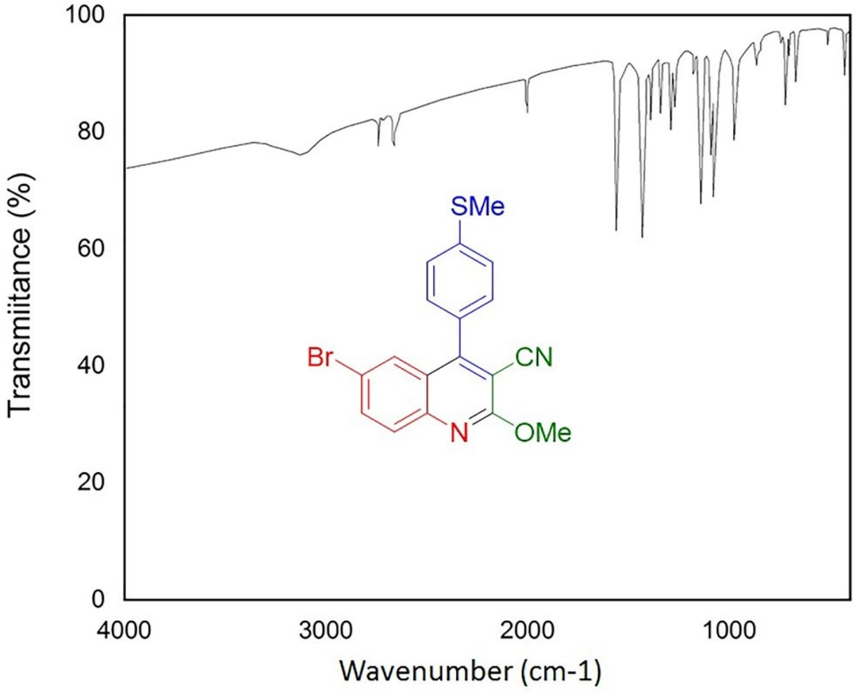

Figure S14. ^{1}H NMR spectrum of compound 4g

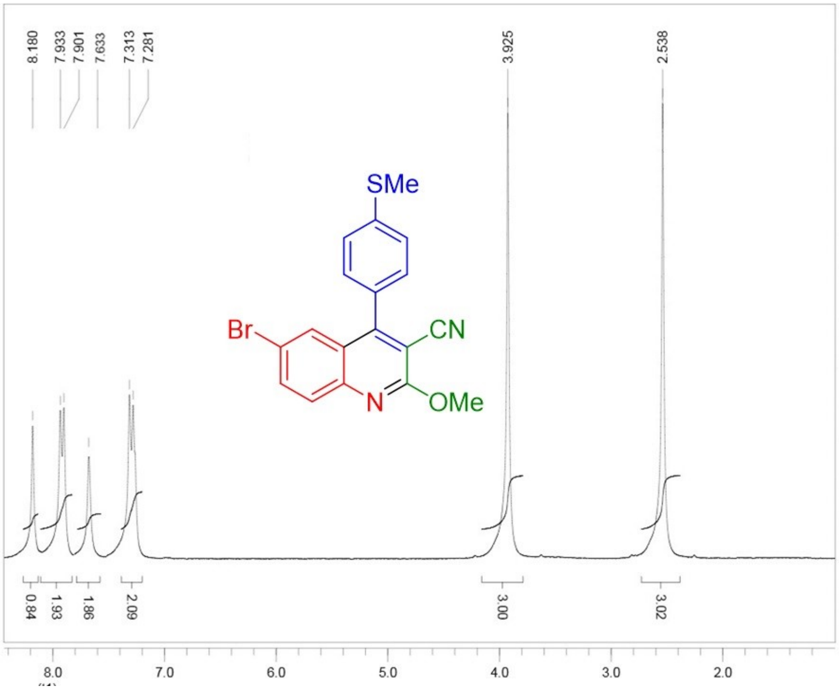

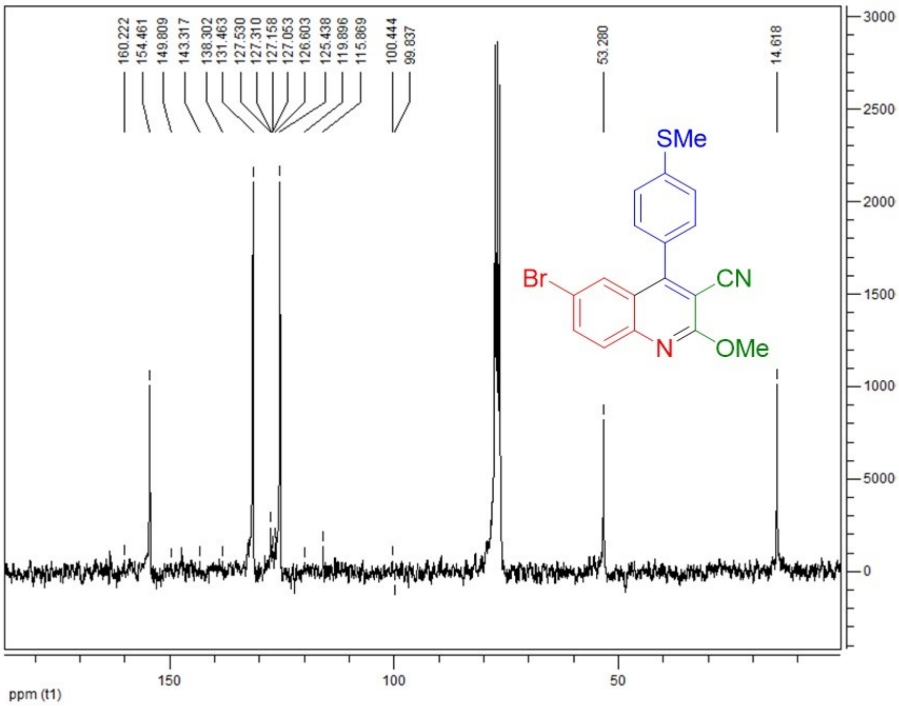

Figure S15. ^{13}C NMR spectrum of compound 4g

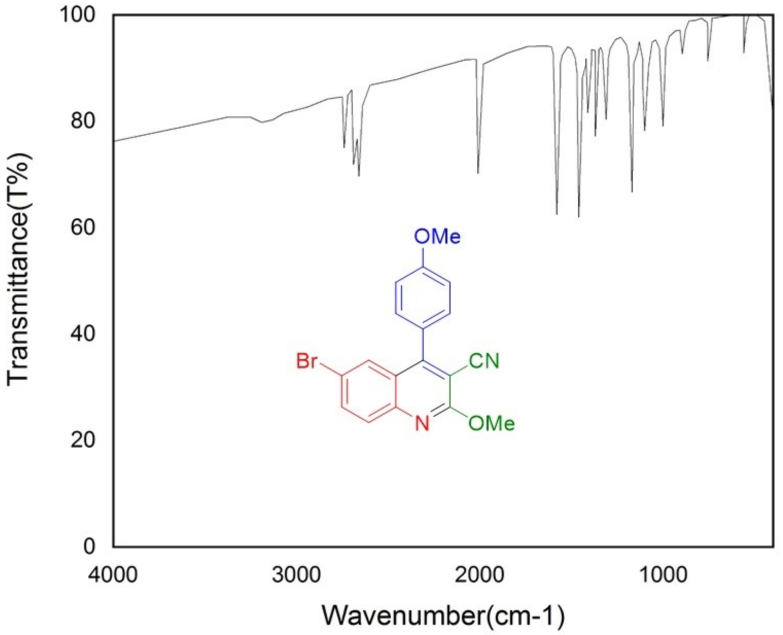

Figure S16. FT-IR spectrum of compound 4h

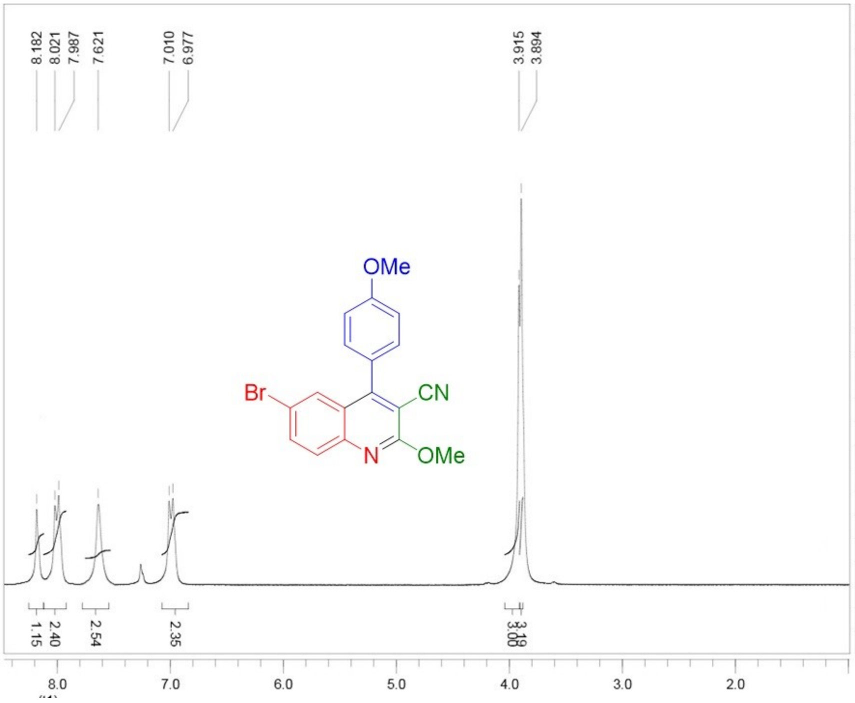

Figure S17. ^{1}H NMR spectrum of compound 4h

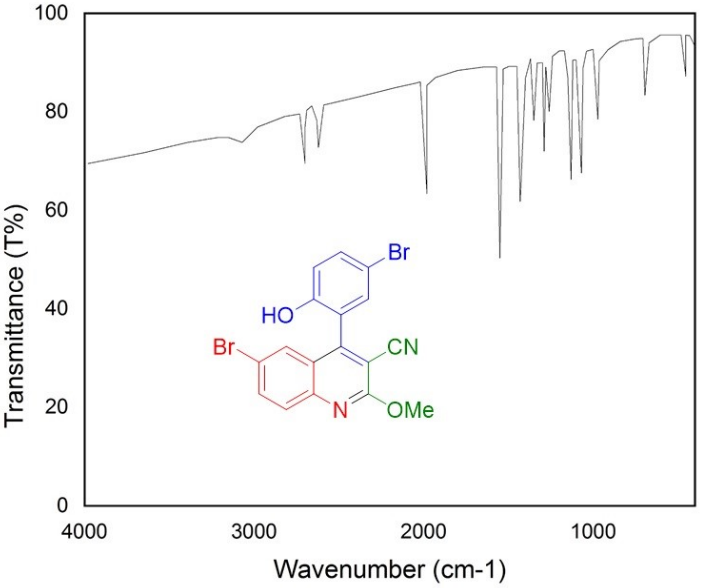

Figure S18. FT-IR spectrum of compound 4i

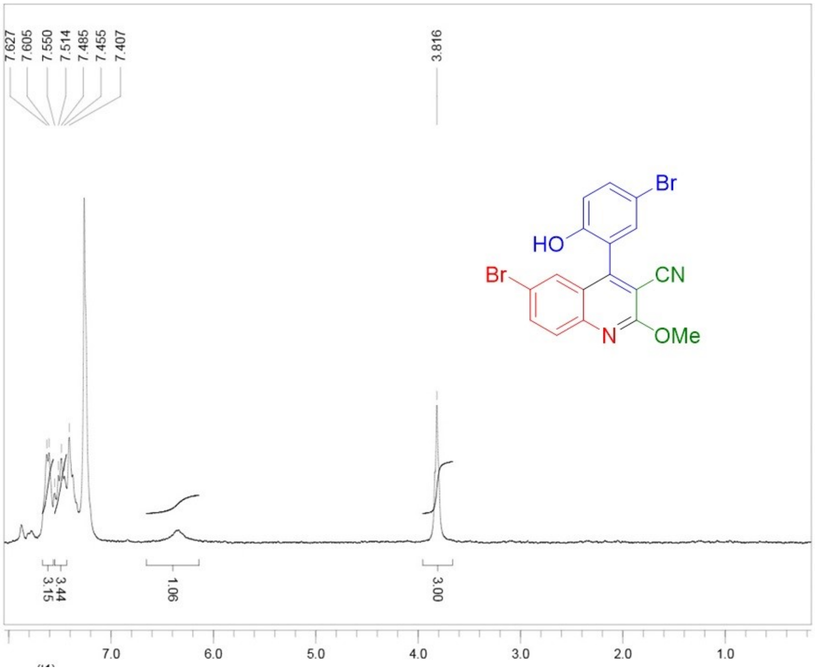

Figure S19. ^{1}H NMR spectrum of compound 4i

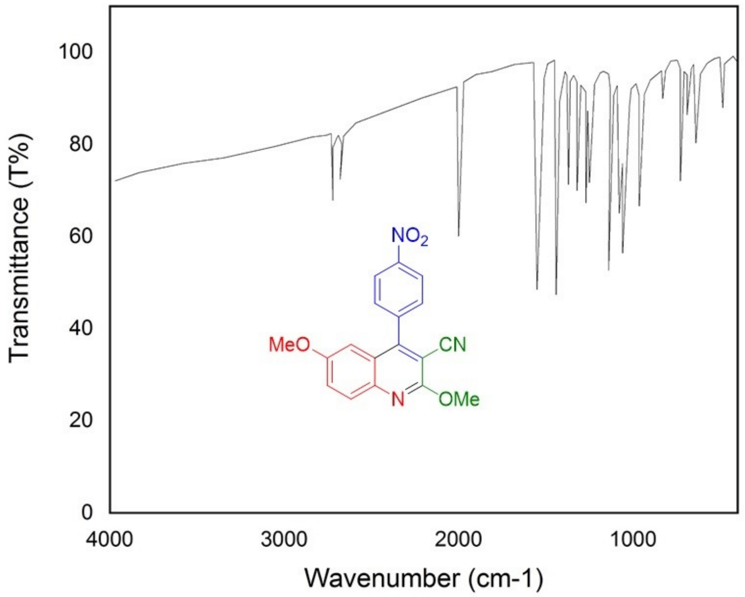

Figure S20. FT-IR spectrum of compound 4j

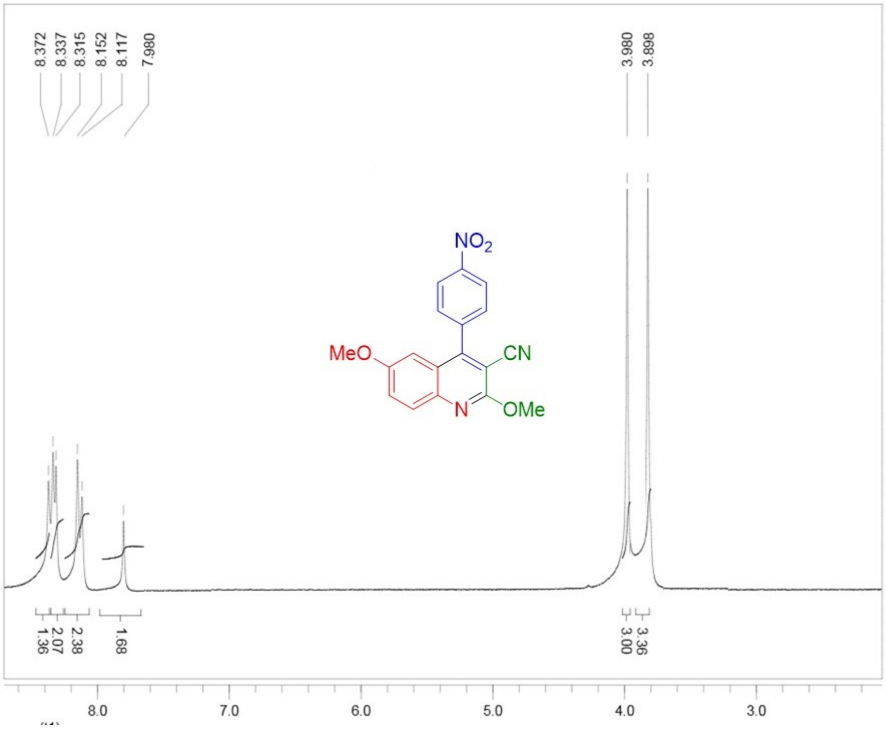

Figure S21. ^{1}H NMR spectrum of compound 4j

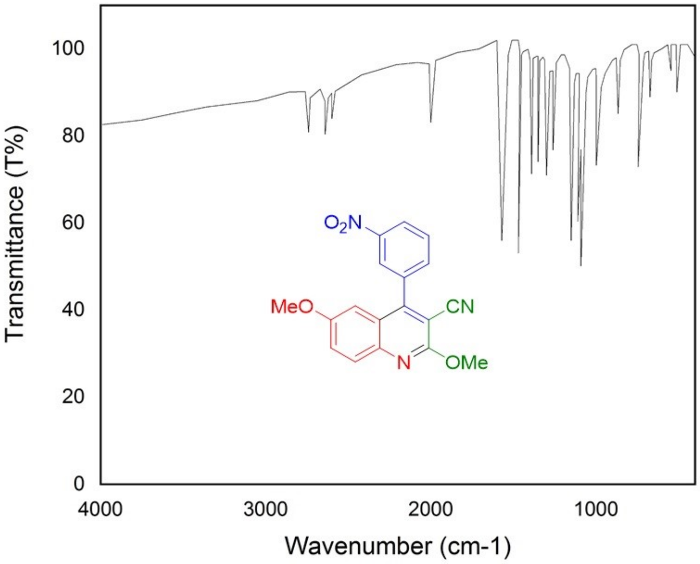

Figure S22. FT-IR spectrum of compound 4k

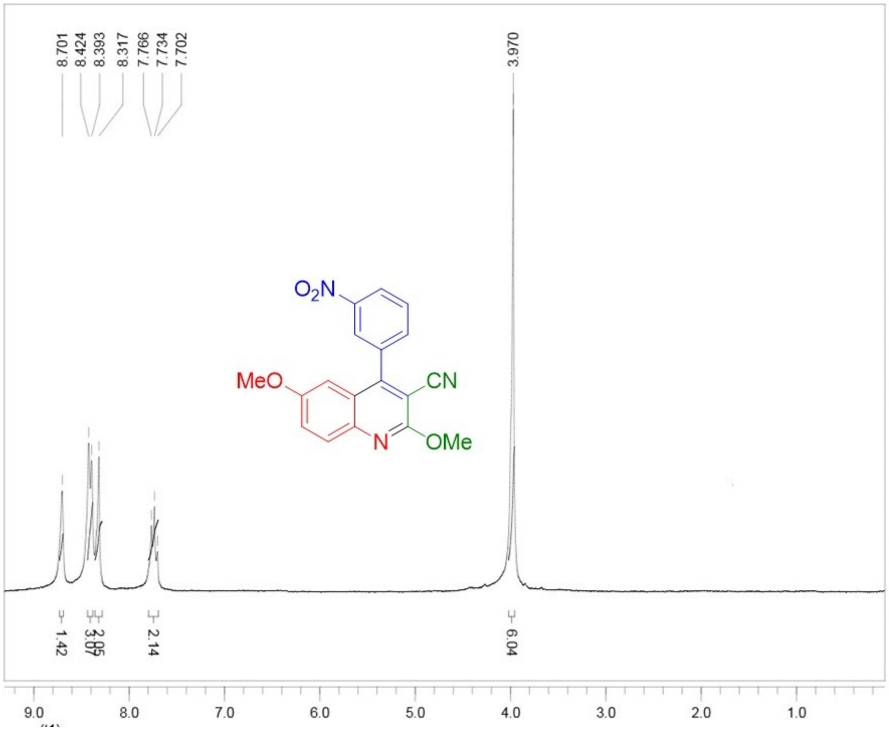

Figure S23. ^{1}H NMR spectrum of compound 4k

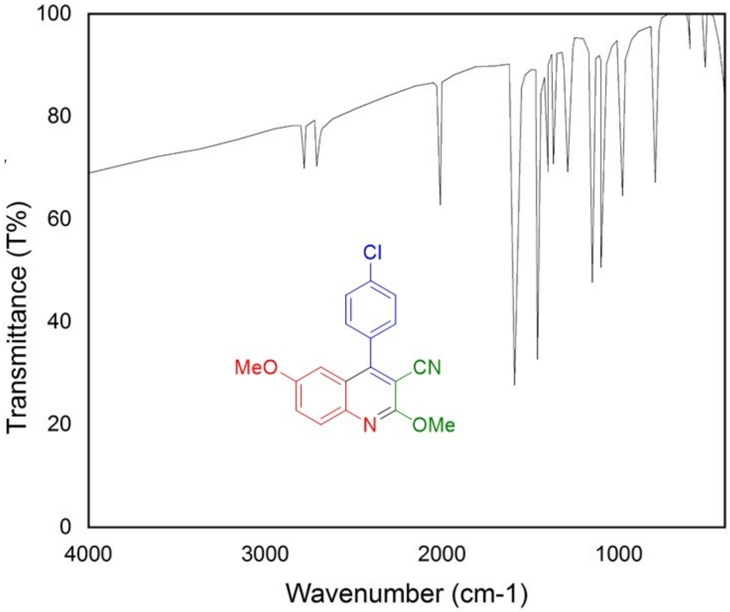

Figure S24. FT-IR spectrum of compound 4l

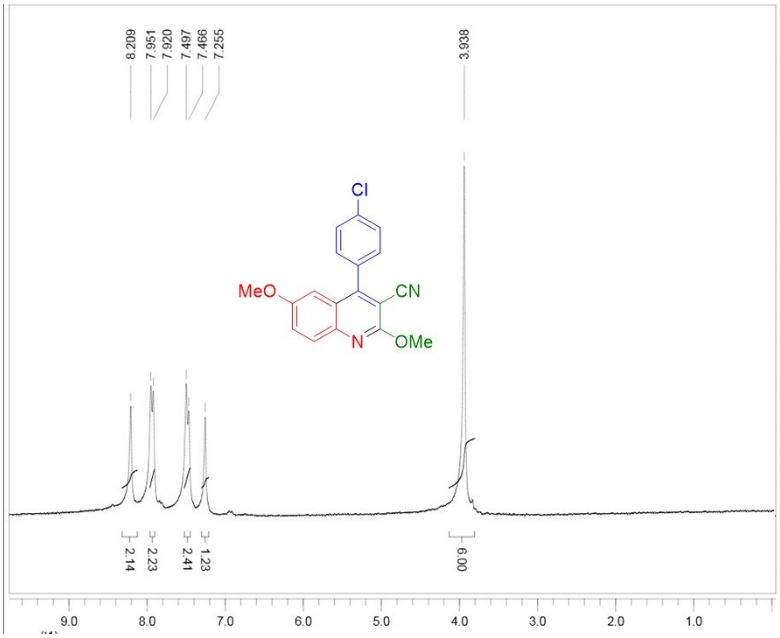

Figure S25. ^{1}H NMR spectrum of compound 4l

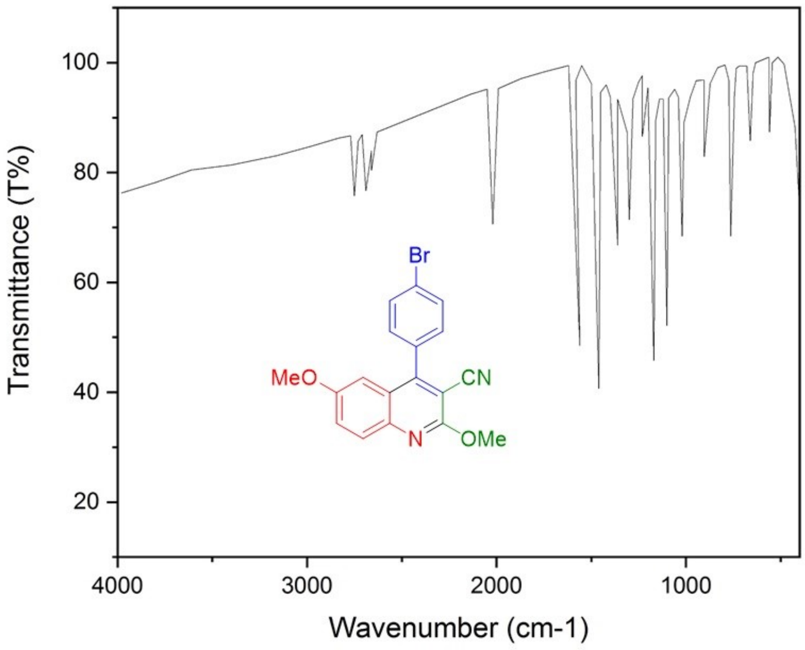

Figure S26. FT-IR spectrum of compound 4m

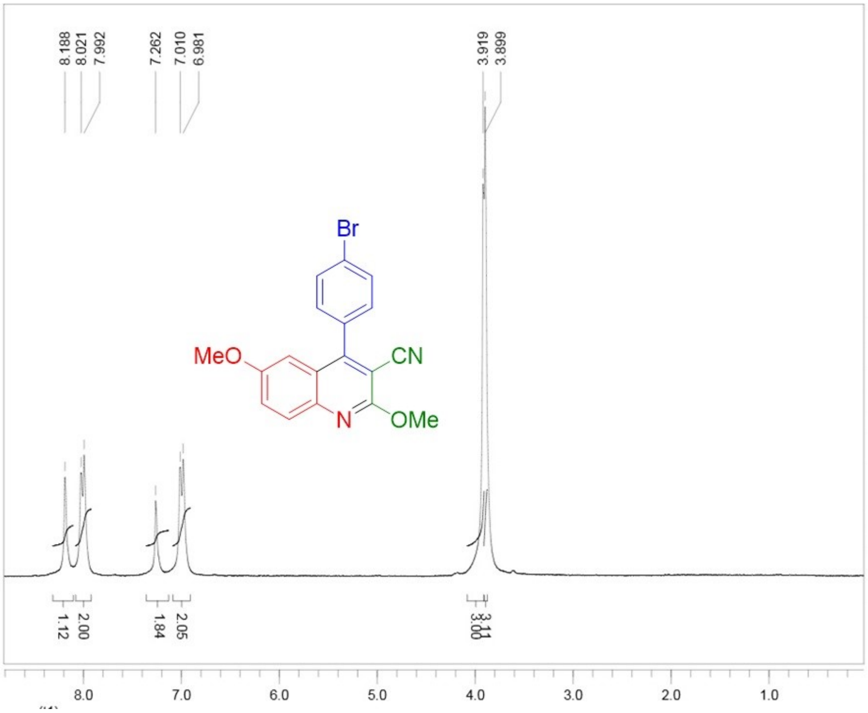

Figure S27. ^{1}H NMR spectrum of compound 4m

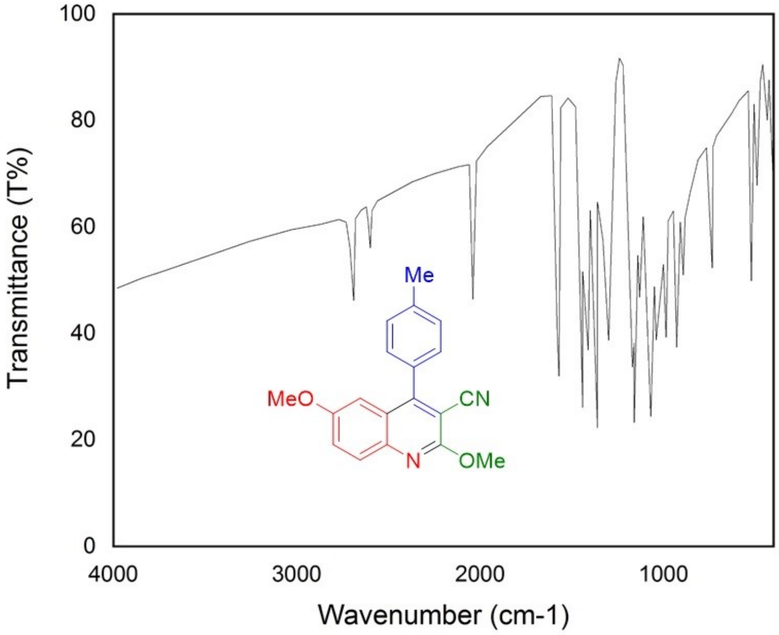

Figure S28. FT-IR spectrum of compound 4n

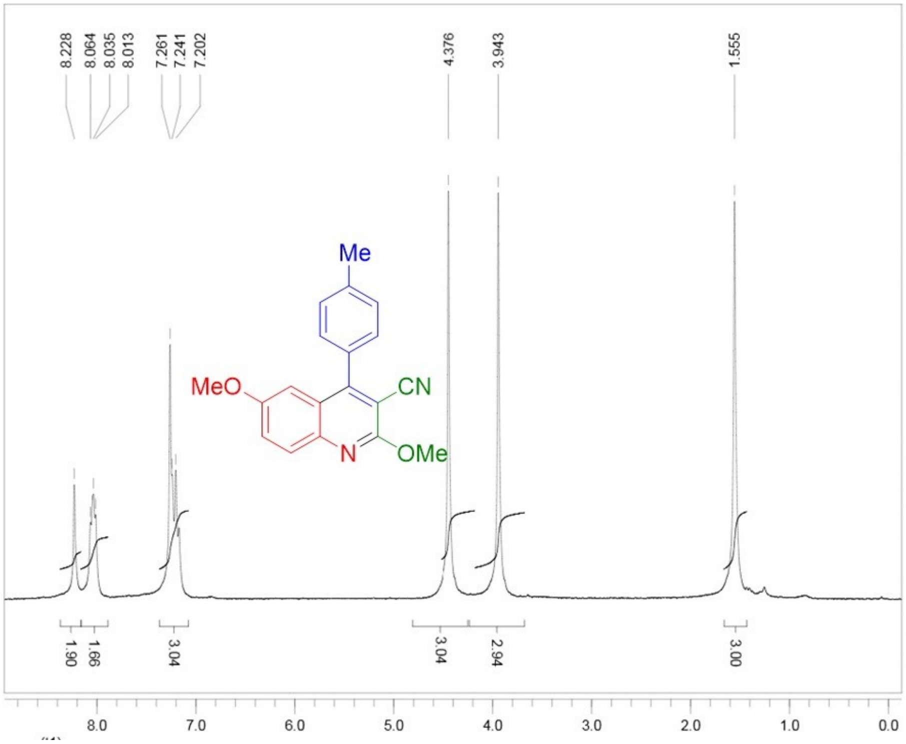

Figure S29. ^{1}H NMR spectrum of compound 4n

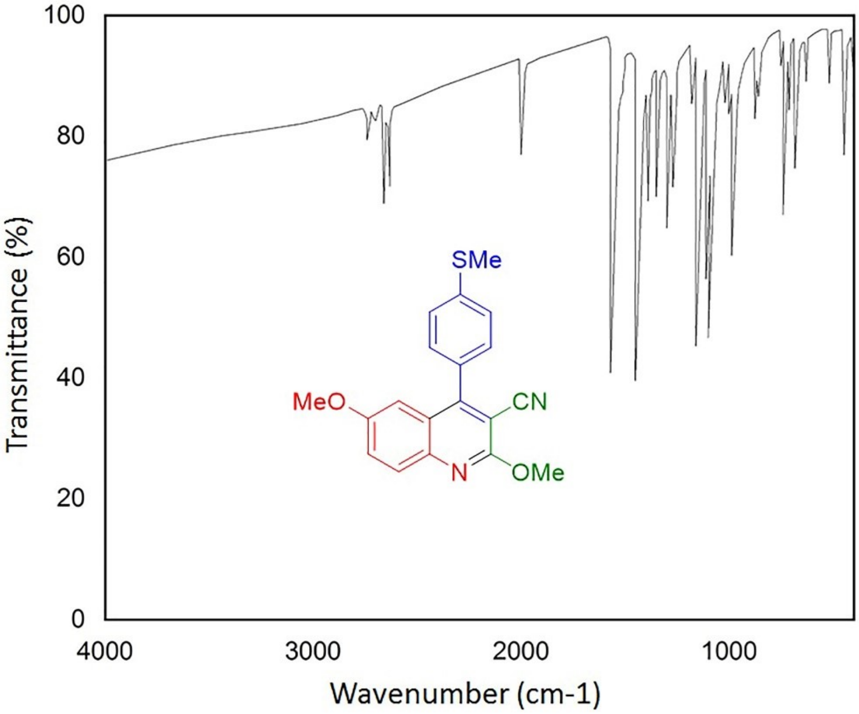

Figure S30. FT-IR spectrum of compound 4o

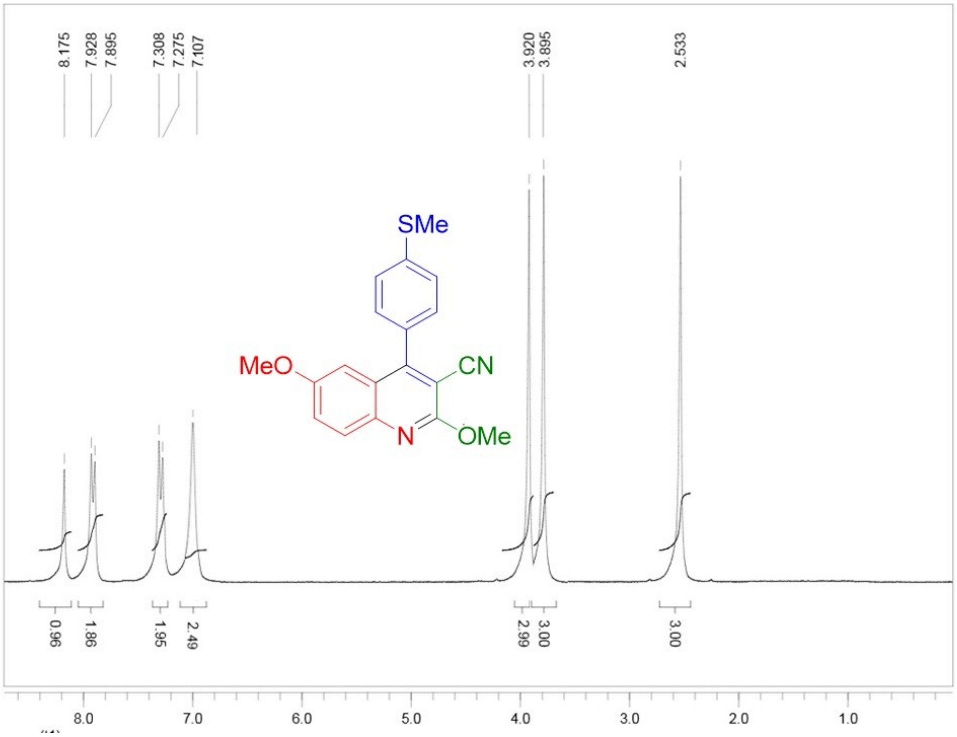

Figure S31. ^{1}H NMR spectrum of compound 4o

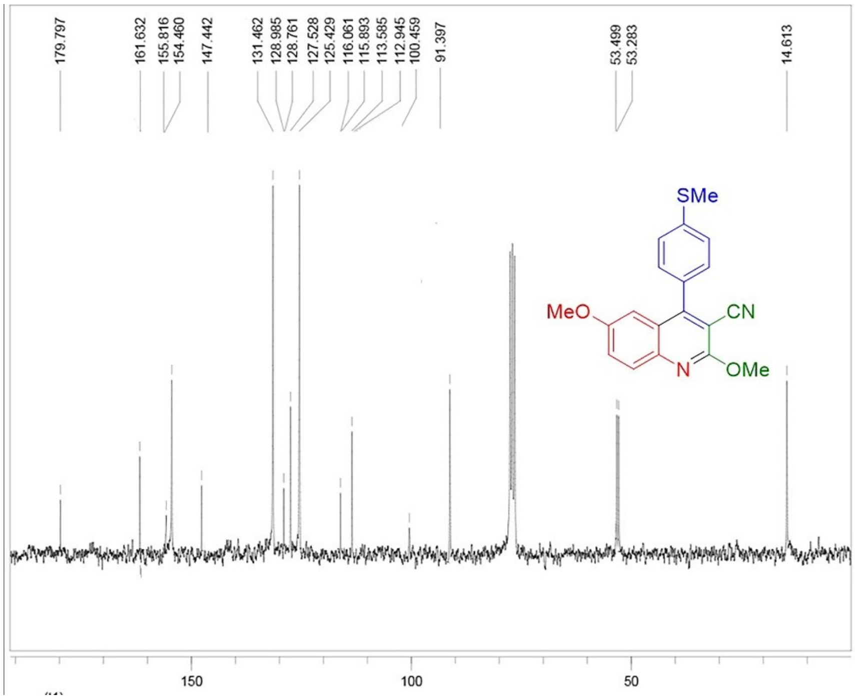

Figure S32. ^{13}C NMR spectrum of compound 4o

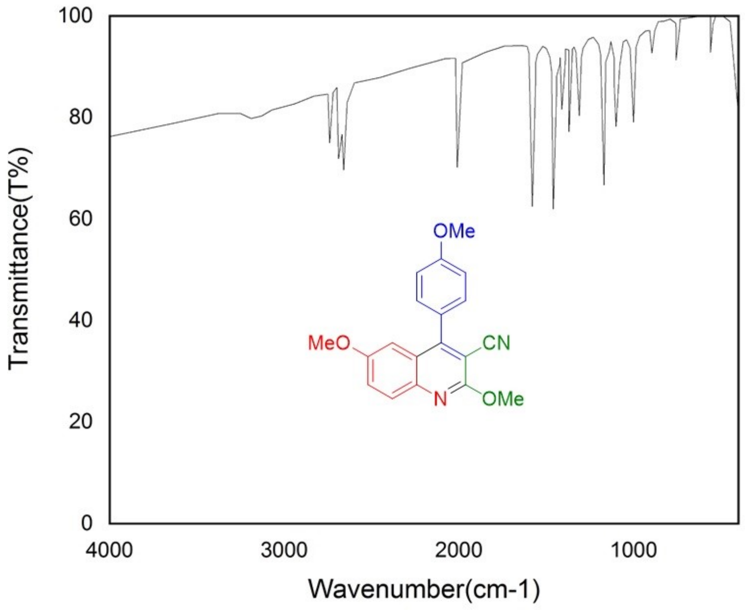

Figure S32. FT-IR spectrum of compound 4p

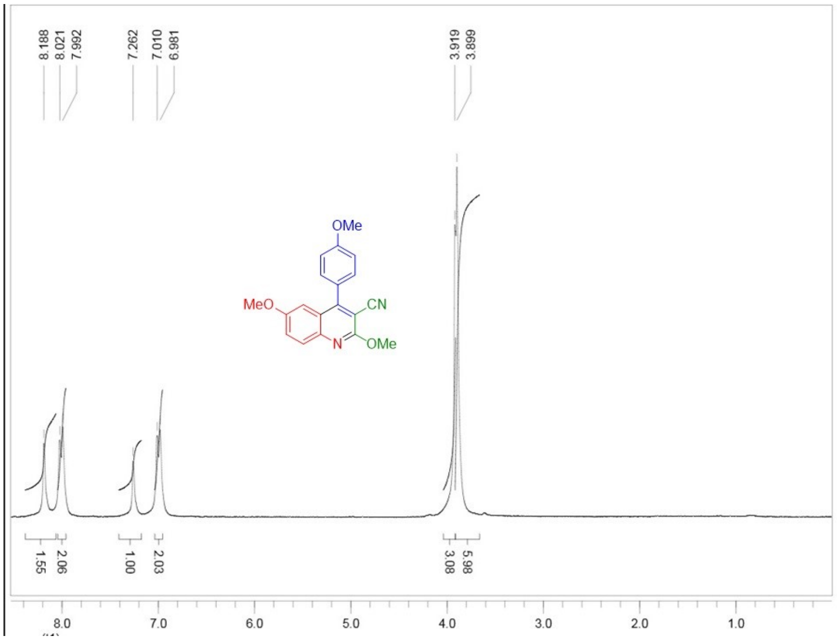

Figure S33. ^{1}H NMR spectrum of compound 4p

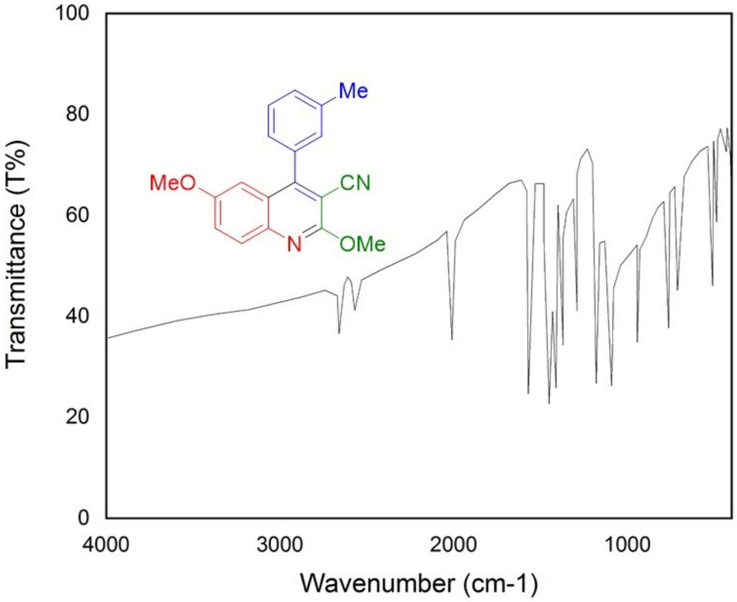

Figure S34. FT-IR spectrum of compound 4q

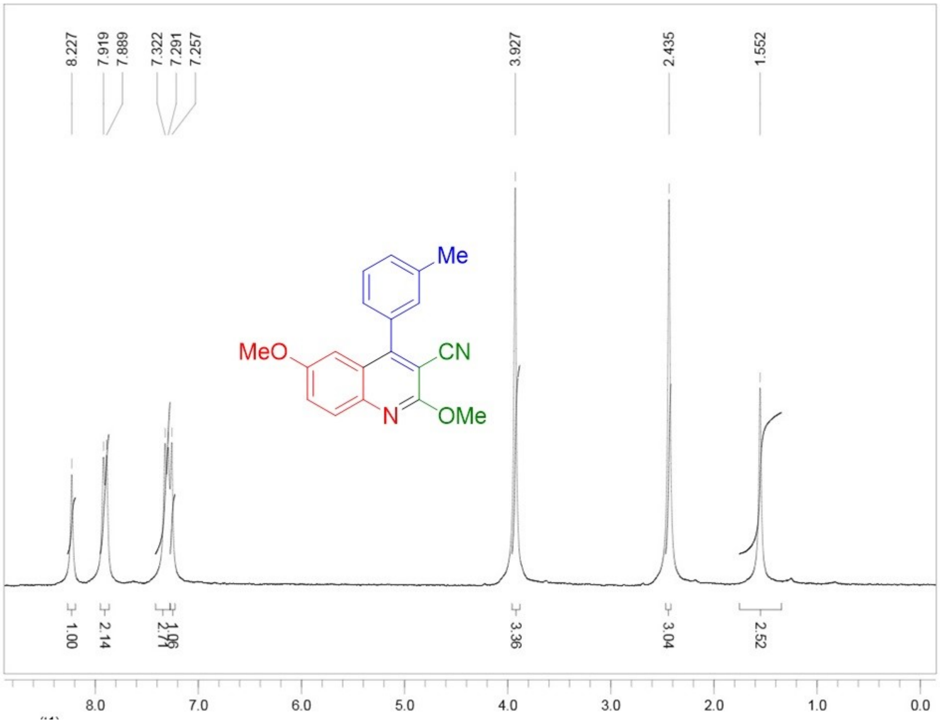

Figure S35. ^{1}H NMR spectrum of compound 4q
